# Involvement of classic and alternative non-homologous end joining pathways in hematologic malignancies: targeting strategies for treatment

**DOI:** 10.1186/s40164-021-00242-1

**Published:** 2021-11-03

**Authors:** Mohsen Valikhani, Elahe Rahimian, Seyed Esmaeil Ahmadi, Rouzbeh Chegeni, Majid Safa

**Affiliations:** 1 Department of Hematology and Blood Banking, Faculty of Allied Medicine, School of Allied Medical Sciences, Iran University of Medical Sciences, Tehran, Iran; 2 Department of Medical Translational Oncology, National Center for Tumor Diseases (NCT) Dresden, Dresden, Germany; 3 Medical Laboratory Sciences, Program, College of Health and Human Sciences, Northern Illinois University, DeKalb, IL USA

**Keywords:** Double-strand break, Double-strand break repair, Non-homologous end-joining, Alternative end-joining pathways, Hematologic malignancies, Targeted therapy

## Abstract

Chromosomal translocations are the main etiological factor of hematologic malignancies. These translocations are generally the consequence of aberrant DNA double-strand break (DSB) repair. DSBs arise either exogenously or endogenously in cells and are repaired by major pathways, including non-homologous end-joining (NHEJ), homologous recombination (HR), and other minor pathways such as alternative end-joining (A-EJ). Therefore, defective NHEJ, HR, or A-EJ pathways force hematopoietic cells toward tumorigenesis. As some components of these repair pathways are overactivated in various tumor entities, targeting these pathways in cancer cells can sensitize them, especially resistant clones, to radiation or chemotherapy agents. However, targeted therapy-based studies are currently underway in this area, and furtherly there are some biological pitfalls, clinical issues, and limitations related to these targeted therapies, which need to be considered. This review aimed to investigate the alteration of DNA repair elements of C-NHEJ and A-EJ in hematologic malignancies and evaluate the potential targeted therapies against these pathways.

## Introduction

**Fig. 1:**
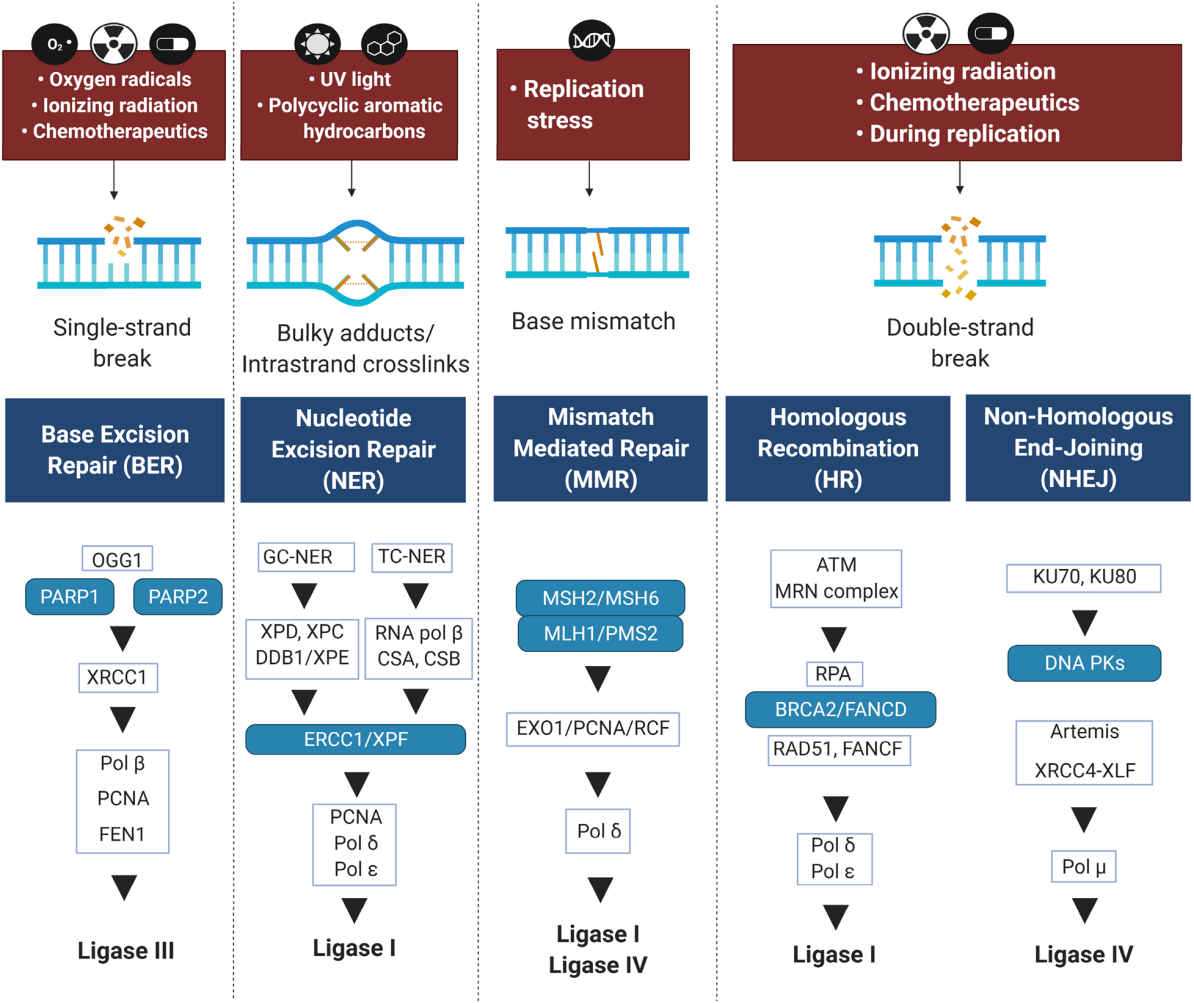
Overview on common causes of DNA damage and the related repair pathways

There are different types of DNA damage including Bulky adducts/intrastrand crosslinks, single-strand break, DNA double-strand break (DSB), and base mismatch (Fig. 1). DSBs are the most destructive genomic damages [1, 2], that may arise either exogenously or endogenously. While the exogenous sources of DSBs include ionizing radiation and DNA damaging agents (clastogens), the endogenous sources commonly result from damages during replication, which, if unrepaired, can stimulate genomic instability [3, 4]. Some mechanisms involved in endogenous DSB formation include V(D)J recombination in progenitors of lymphocytes, class-switch recombination (CSR) in lymphocytes, and meiosis followed by gametogenesis [5].

On the other hand, DSB genotoxicity can be compensated by two major pathways: (1) homologous recombination (HR); and (2) non-homologous end-joining (NHEJ), including classical non-homologous end-joining (C-NHEJ) and alternative non-homologous end-joining (A-NHEJ or A-EJ) pathways [5]. The NHEJ pathways rejoin two broken DNA ends and repair DSBs in G1 or G0 phase of the cell cycle. While recognition of DNA ends by C-NHEJ pathways is dependent on XRCC5, XRCC6, DNA-PKcs, and ligation by DNA ligase IV (Lig IV)/XRCC4, the A-EJ pathways are independent of Lig IV and can recognize DNA ends by a diverse set of factors, including different DNA polymerases (δ and θ), DNA nucleases (ERCC1-XPF), and ligases (Lig I and Lig III/XRCC1) [6].

Aberrant repair of DSBs can result in miss-joining of DNA repair components with DNA ends and cause deletions, inversions, or complex rearrangements of chromosomes. These changes all lead to genomic instability, tumor susceptibility, immunodeficiency, and a wide range of human cancers, including hematologic malignancies [6–8]. Genomic instability due to the aberrant activity of NHEJ pathways can also increase the ratio of acquired mutations or translocations, including recurrent translocations in hematologic malignancies, such as BCR-ABL and MLL translocations, as the most important ones [9–11].

Several studies have shown that chemo- or radio-resistant leukemic cells have altered levels of C-NHEJ and A-EJ activities, compared to their sensitive counterparts. Considering the development and progression of hematologic malignancies via DNA damage and repair response abnormalities, it seems that use of DSB inducers, in combination with DSB repair (DSBR) inhibitors, may be a promising strategy to eradicate malignant cells and provide a novel therapeutic approach. Therefore, this study aimed to investigate the role of C-NHEJ and A-EJ pathways in the progression of hematologic malignancies and to evaluate targeting of these pathways for reducing the mortality of patients.

### Mechanisms of C-NHEJ and A-EJ pathways in DSBR

**Fig. 2:**
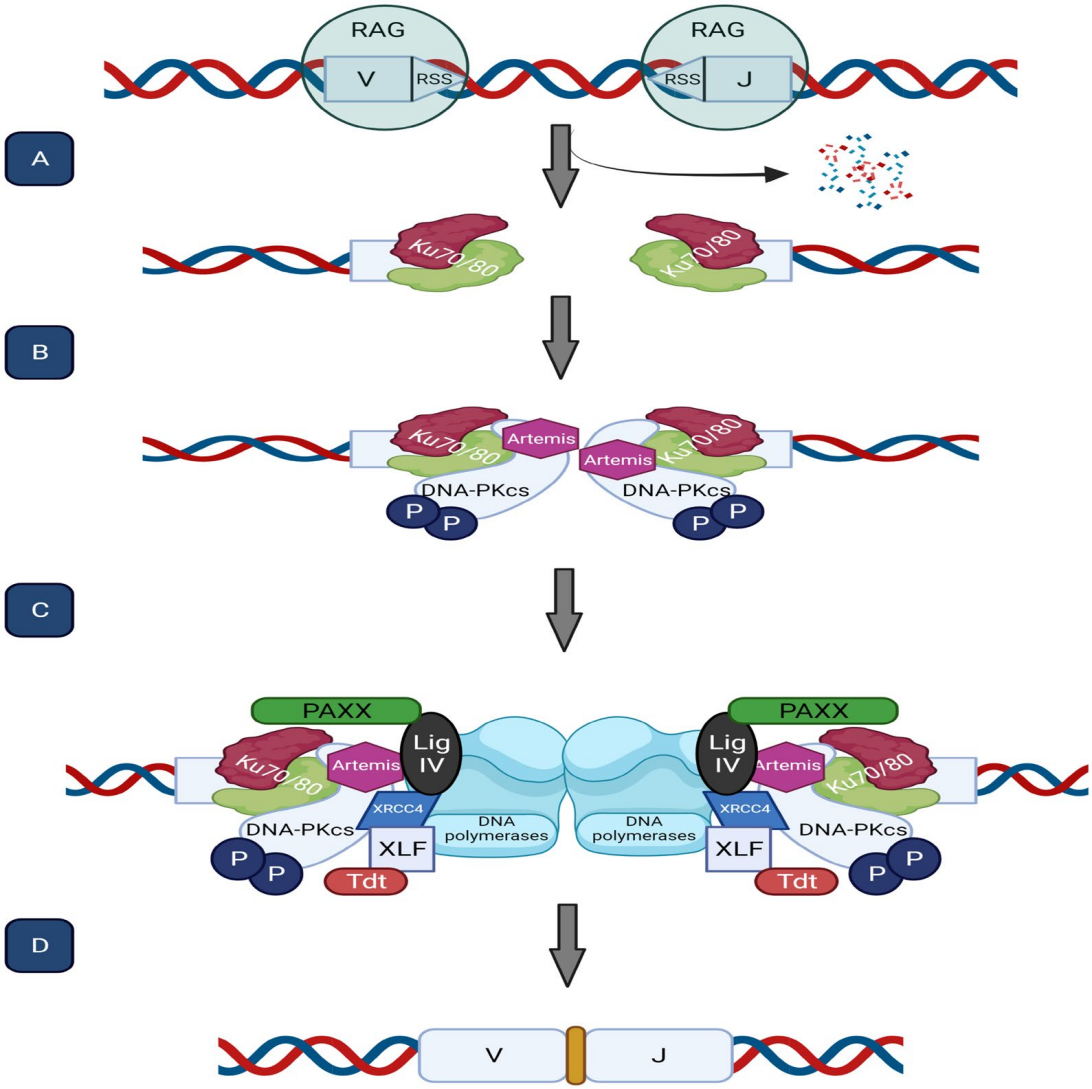
C-NHEJ in V(D)J recombination process. **A** RAG proteins bind to the V(D)J sequence at DNA, leading to DNA cleavage, creating DSB. Afterward, Ku70/80 heterodimers locate and bind to the DSB end. **B** Ku70/80 directly recruits DNA-PKcs. Following DNA-PKcs autophosphorylation, it activates the main nuclease in c-NHEJ, Artemis. **C** After trimming of DNA ends by Artemis, DNA polymerases reconstruct the DNA. Consequently, the Ku-DNA complex anchors PAXX, XRCC4, XLF, and Lig IV to rejoin the DNA ends. D Fully functional recombined DNA is ready to be translated

In human cells, C-NHEJ is a rapid, high-capacity pathway that mediates the direct religation of the broken DNA molecule with minimal reference to the DNA sequence. In contrast to HR, C-NHEJ does not require an extensive homologous template; therefore, it is more error-prone and theoretically is not restricted to a certain cell cycle phase [12]. The mechanism of C-NHEJ can be broken down into several sequential steps. The initial step is the recognition and binding of the Ku70–Ku80 (also known as XRCC6–XRCC5) heterodimer to the DSB. Ku heterodimer serves as a ‘tool belt’ or a scaffold that directly or indirectly recruits other NHEJ proteins [13]. As an essential event, Ku70/80 directly recruits DNA-dependent protein kinase catalytic subunit (DNA-PKcs) to the DNA ends. DNA-PKcs has a strong affinity for Ku–DNA ends and, together with Ku, form the DNA-PK complex. Following the binding of DNA-PKcs to the DNA-Ku complex, the Ku heterodimer translocates inward on the dsDNA strand and eventually results in serine/threonine protein kinase activation of the DNA-PKcs [14]. DNA-PKcs undergoes autophosphorylation and activates Artemis, the main nuclease in NHEJ, which then gains the ability to trim overhangs to expose complementary regions. The trimming of different end structures such as DNA loops, flaps, or gaps by Artemis makes them suitable for the ligation of the XRCC4–LIG IV complex (Fig. 2) [13, 15].

For more complex ends, other factors (e.g., PNKP, APTX, APLF, and PALF) and polymerases (pol μ and pol λ) are required [16, 17]. To ligate the broken ends, the Ku-DNA complex anchors PAXX, XRCC4, XLF (NHEJ1 or Cernunnos), and Lig IV, rejoining the DNA ends [18].

### A-EJ components and mechanisms

In mammalian cells, the repair of DSBs by A-EJ is more evident in the absence of a functional C-NHEJ pathway [19, 20]. There is an increasing interest in A-EJ pathways in malignant cells, as they create large deletions, translocations, and genomic rearrangements [21–23]. Therefore, they might serve as promising therapeutic targets in tumor cells with deficiencies in main DSB repair pathways. These pathways are Ku-independent and require DNA end resection, similar to HR. Since the broken ends can be rejoined without using a homologous template, this process also shares similarities with NHEJ [24]. Based on the amount of DNA sequence homology used to align DNA ends, the A-EJ mechanisms are mediated by two minor pathways: single-strand annealing (SSA) and microhomology-mediated end-joining (MMEJ) [25]. While SSA comprises complementary repeat sequences greater than 25 nucleotides, MMEJ involves microhomologies which are shorter tracts of sequence homology (2–20 nucleotides) [26].

Several studies have shown that PARP1 binds to single-strand DNA and is essential for the initial phase of A-EJ (recognition and tethering). First, it catalyzes the poly-ADP-ribosylation of proteins at DNA damage sites [27]. Next, it contributes to the initial assembling of the MRN complex (including MRE11, RAD50, and NBS1) on DSBs, leading to the activation of ataxia telangiectasia mutated (ATM) and RAD3-related (ATR) kinases [28]. This complex causes DNA end resection, which involves two major steps. In the first step, the combination of MRN and C-terminal interacting protein (CtIP) creates short single-stranded DNA (ssDNA), and then exonuclease 1 (EXO1) or Bloom’s helicase (BLM)/DNA2 endonuclease complex causes an extensive end resection [29]. EXO1 is loaded on ssDNA by Metnase (or SETMAR), a chimeric fusion protein consisting of a transposase domain and a histone methylase domain; the former is MAR, and the latter is called SET [30]. Metnase enhances DSBR through the C-NHEJ pathway by interacting with DNA Lig IV [31]. Also, Metnase and Artemis nucleases determine the fidelity of end-joining repair in mammalian cells (Fig. 2) [32].

**Fig. 3:**
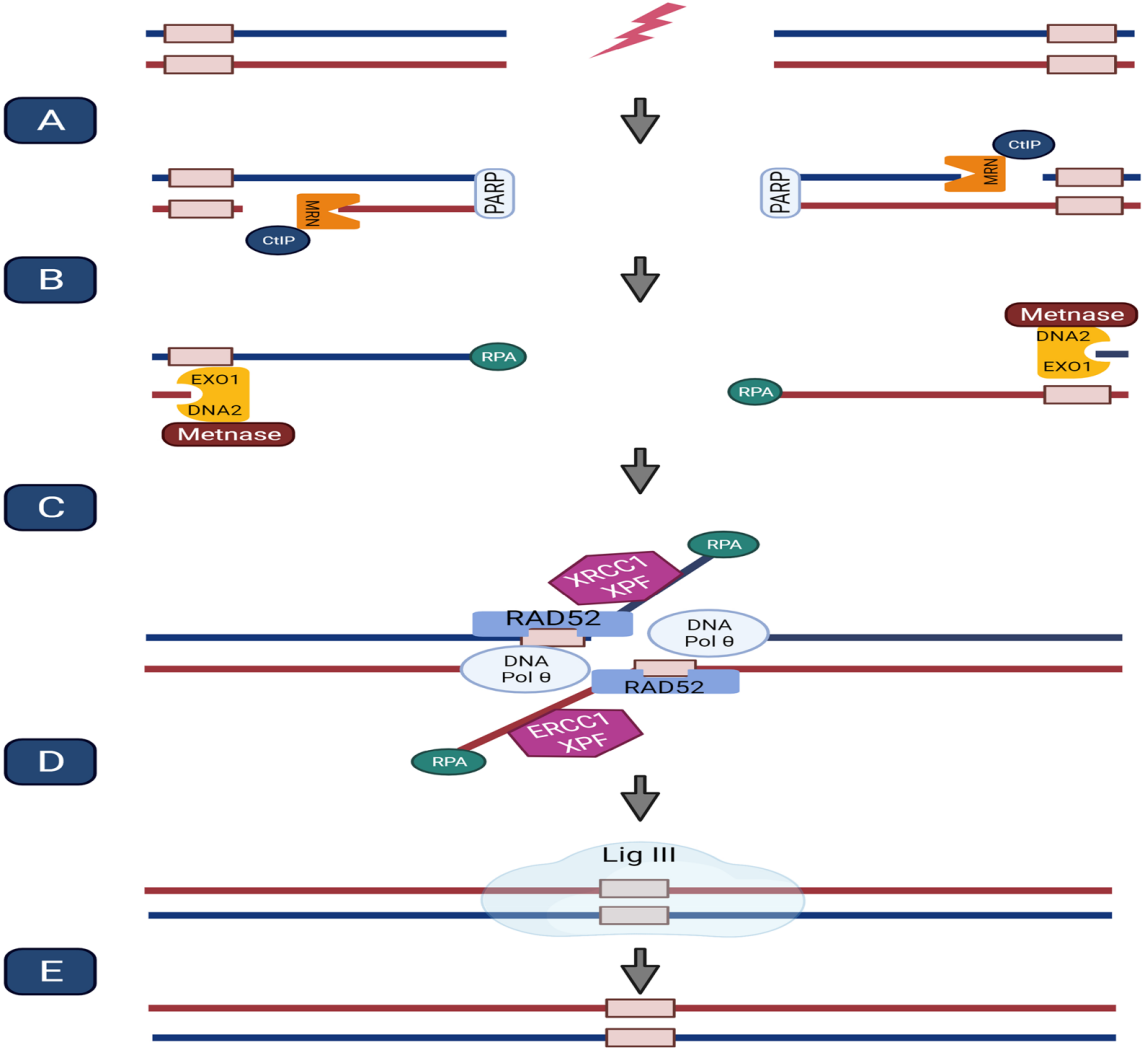
Alternative non-homologues end joining pathway. **A** PARP1 binds to single-strand DNA for the recognition of damages. It catalyzes the poly-ADP-ribosylation of proteins at DNA damage sites. Also, it contributes to the initial assembling of the MRN complex on DSBs, leading to the activation of ATM and ATR kinases. **B** In SSA, the combination of MRN and CtIP creates short ssDNA, and then, EXO1 or BLM/DNA2 endonuclease complex causes an extensive end resection. EXO1 is loaded on ssDNA by Metnase. **C** RAD52 binds to the RPA-coated single strand annealing the complementary regions. ERCC1/XPF complex removes the tails. This step of DNA end resection is dispensable for MMEJ. **D** Then, polymerases, flap endonucleases, helicases, and polynucleotide kinases prepare the DNA ends for ligation (the gap-filling component of SSA is unidentified). **E** Finally, Lig III ligates the DNA ends with the help of XRCC1 as a scaffolding protein

The second step of DNA end resection is dispensable for MMEJ [26]. Polymerases, flap endonucleases, helicases, and polynucleotide kinases prepare the DNA ends for ligation [5]. Pol θ fills the gap in MMEJ, whereas the gap-filling component of SSA is unidentified [26]. Finally, Lig III ligates the DNA ends, although other components, such as XRCC1, as a scaffolding protein, are needed [33]. It should be noted that interlinking issues are one of the important factors in the repair process and selection of a pathway, as well as targeted therapy. Overall, neddylation, ubiquitination, and interference of non-coding RNAs are the most common interlinking issues in DSBR [34–36]. Moreover, the mechanism of the A-EJ pathway is shown in Fig. 3.

### C-NHEJ and A-EJ alterations in hematologic malignancies

Hematologic malignancies have been at the forefront of cancers in terms of using genetic analyses for diagnosis, classification, prognosis, and clinical therapeutic management of patients. Genomic analysis has dramatically influenced the clinical evaluation of nearly every form of hematologic malignancy. DNA repair has a critical role in protecting cells against endogenous or exogenous insults that can cause varying degrees of DNA damage. Any deficiency in DNA repair pathways results in various genomic changes that ultimately may give rise to tumorigenesis and the development of hematological malignancy [37]. Here, alterations in C-NHEJ and A-EJ components are separately discussed in four categories: leukemia, lymphoma, myelodysplastic syndromes (MDS), and multiple myeloma (MM).

#### Leukemia

Genomic instability is one of the key drivers of hematological malignancy and is responsible for leukemia progression [38]. Genomic instability, including mutations in DNA sequences, chromosomal aneuploidy, translocations, and gene amplifications, are frequently found in leukemia cells suggesting that the DSB response may be altered. A growing body of evidence showed that dysregulation of DSB repair pathways could predispose patients to different leukemia. Deficiencies in DNA repair pathways are causal factors for many solid cancers, but they are only just beginning to be explored in leukemia. Here, changes in the DSB repair pathway in leukemia, including acute lymphoblastic leukemia (ALL), acute myeloid leukemia (AML), chronic lymphocytic leukemia (CLL), and chronic myeloid leukemia (CML) are described in detail.

#### CLL

Pathogenesis of CLL is characterized by specific genetic abnormalities and changes in cellular signaling pathways. In particular, a disrupted DDR plays the main role in increasing CLL cell survival. Many studies assessed the expression of genes involved in the repair pathway to test how the DSB-repair deregulations are involved in the initiation and progression of the CLL. The elevated levels of MMEJ factors have been observed in B-CLL cells and it was concluded that CLL-specific increased expression levels of the MMEJ factors Lig I and XRCC1 associated with an increased chance of gaining chromosomal aberrations throughout DSBR [6]. Klein et al. assessed the associations between the expression levels of proteins regulating apoptosis (BCL-XL and BCL-2) and DNA repair in B-CLL cells and normal B cells. They found a close relationship between Bcl-xL and Bcl-2 expression and Ku80 levels suggesting that in B-CLL cells, modulators of the apoptosis and DNA repair are regulated in a coordinated manner [39]. In another study, CLL cells demonstrated a significantly lower frequency of cells staining positive for DNA-PKcs and Ku86, but not for Ku70, in comparison with ALL cells. Surprisingly, MM samples were reported to express significantly higher DNA-PKcs, Ku86, and Ku70 protein levels compared to CLL. Therefore it was suggested that DNA-PK expression coincides with the degree of lymphoid malignant cells maturity [40]. DNA-PKcs was also shown to be overexpressed in CLL patients with del(17p) and del(11q), indicating that DNA-PK may contribute to disease progression. Moreover, these data support the hypothesis of targeting DNA-PKcs in poor-risk CLL and demonstrate a validation for the use of a DNA-PK inhibitor [41].

Analysis of DNA-binding activity of the Ku70/80 heterodimer showed an increased DNA-binding activity in the resistant B-CLL cells compared to the sensitive cells (before and after irradiation treatment). Elevated levels of DNA end-binding by the Ku70/Ku80 heterodimer up-regulate DNA-PKcs and NHEJ activity and facilitate the escape of resistant B-CLL cells from apoptosis even in the presence of irradiation-induced DNA damage [42].

Accumulation of DNA damages and error-prone DNA repair are critical features of genetic instability that are believed to be involved in the pathogenesis of CLL [43]. Although the role of ATM in signaling to repair proteins is associated with a function that could result in resistance mechanism against the alkylating agents, unexpectedly, the loss of ATM protein is consistent with a poor prognosis and aggressive disease in CLL. Austen et al. analyzed 155 CLL cases for ATM mutations, and they found that two-thirds of the medically treated patients with ATM mutations were clinically refractory to DNA damaging chemotherapeutic drugs. A hypothesis suggests that, as ATM can act upstream of p53 in response to DSB to stimulate cell cycle arrest and apoptosis, loss of ATM mitigates the p53-dependent cell death, resulting in a chemo-refractive phenotype [44].

Moreover, telomere length is a prognostic indicator in CLL patients. Short-dysfunctional telomeres can cause illegitimate end-to-end telomeric fusions of chromosomes, leading to genomic instability and disease progression in CLL. A recent study has elucidated the role of C-NHEJ and A-EJ in mediating telomere fusions and suggested that therapeutic agents targeting these DNA repair pathway factors may efficiently sensitize CLL B-cell clones with telomere dysfunction to improve outcomes in patients [45].

#### ALL

ALL is the most common childhood leukemia and the foremost cause of childhood tumor deaths. During recent decades the occurrence rate of ALL has grown around 30%, whereas the age-standardized incidence rate has stayed almost unchanged. Among all risk factors, smoking has been found to be the chief factor contributing to mortality of ALL cases, therefore, avoiding exposure to carcinogens is of a great importance. Moreover, the high body mass index is another critical factor role-playing in ALL patients’ death [46]. Although most pediatric ALL patients respond well to chemotherapy, the outcome becomes less favorable when patients relapse. Cytogenetic alterations are common, and some molecular markers have been recognized to predict the prognosis [47, 48]. Researches revealed that chromosomal translocations that appear prenatally are the primary event in multistage leukemia development. These translocations give rise to gene fusions, such as BCR-ABL and TEL-AML1, which generate altered proteins. Alterations of DNA repair pathways have also been examined in ALL. Using a sensitive approach that is based on automated enumeration of DSB co-localizing proteins γH2AX and 53BP1, a higher γH2AX/53BP1 foci were detected in ALL patients harboring BCR-ABL or TEL-AML1 than patients without gene fusions, suggesting that BCR-ABL/TEL-AML1 induces DNA instability through facilitating further genetic alterations which drive leukemogenesis [49].

AT is a cancer-predisposing disease that individuals are born with two mutated copies of the ATM gene. Patients develop mostly, leukemia and lymphoma. A higher prevalence of chromothripsis (several clustered chromosomal rearrangements in one or few chromosomes) was reported in the genomic landscape of ALL arising in individuals with AT, probably due to the related deficiency in ATM mutation [50]. Similar to AT syndrome, Nijmegen breakage syndrome (NBS) is a cancer-predisposing disease of childhood, resulting from mutations in the NBS1 protein of the MRN complex. Children with NBS usually have concomitant hematologic malignancies, including ALL, T-cell prolymphocytic leukemia (T-PLL), and non-Hodgkin lymphoma (NHL) [51–54]. Mutations in Lig IV, which was associated with reduced and less proficient NHEJ, have also been reported in ALL patients [55]. In both murine and human T-ALL cells, the incidence of KRAS mutations associate with the increased expression of A-EJ factors, including DNA Lig IIIa, PARP1, and XRCC1 [56].

Some studies report a correlation between upregulated DNA repair and the stage of the disease in ALL. Using Real-time PCR, Chiou et al. assessed the mRNA transcript of some NHEJ members, including Ku70, Ku80, DNA-PK, Artemis, XRCC4, Lig IV, and Cernunnos/XLF in pediatric ALL patients at different phases of the disease. Compared to thalassemia patients, which were considered control samples in this study, the mRNA expressions of all NHEJ factors were elevated in untreated fresh ALL. After the therapy and once patients achieve complete remission, overexpressed NHEJ mRNAs were downregulated. However, mRNA expressions of Ku80, DNA-PK, Artemis, XRCC4, and DNA ligase IV were raised again in relapsed cases [57]. In another study, only 22% of adult ALL patients with high Ku80 expression achieved durable complete remission compared with 62% of low expresses, suggesting that Ku80 might contribute to poor prognoses in adults with ALL [58].

Polymorphisms in DNA repair genes may modify protein function and cell's capability to repair damaged DNA. There seems to be a correlation between childhood leukemia and a specific polymorphism in the XRCC6 promoter (T-991C). Previous studies have shown that patients harboring TC genotypes are predisposed to a higher risk of childhood leukemia compared to those harboring TT wild-type genotypes [59]. Similarly, XRCC1 (Arg194Trp) polymorphism increases the risk of leukemia. However, the outcomes are different in various studies. For instance, an increased risk of childhood ALL was reported in an Egyptian population, especially in females [60]. In contrast, no association was found between XRCC1 polymorphisms and increased risk of ALL in a Mexican pediatric population [61]. In patients who developed therapy-related-acute promyelocytic leukemia (t-APL) following mitoxantrone treatment of multiple sclerosis (MS), a marked linkage with 1572G > A polymorphism in *XRCC5* gene has been observed [62]. It is noteworthy that homozygous variants of BRCA2 and XRCC5 are associated with a greater risk of secondary acute promyelocytic leukemia (APL). Likewise, some polymorphisms in both *XRCC5* and *XRCC6* genes increased the risk of leukemia in a Chinese population [63].

#### AML

AML is the most common adult acute leukemia with variable prognosis, based on the cytogenetic features. The occurrence rate of AML exhibits an increasing pattern, in which males and elderly people are the most probable cases to develop AML. Regarding age among AML patients, a comparison of developing and developed countries betokened a higher mortality rate in the latter [64]. AML is classified as a heterogeneous clonal neoplasm in which different translocations and mutations are involved. Moreover, recurrent mutations in genes such as FLT3, TP53, CEBPA, NPM1, RUNX1, IDH1/2, DNMT3A, KMT2A, and ASXL1 exacerbate the burden of the disease [65]. Given the increased incidence of AML, targeted and effective therapeutic approaches are required to lower the burden of this disease.

Genetic and epigenetic changes can trigger aberrant DNA damage response in AML cells and induce disease progression and resistance to chemotherapy [66, 67]. Many research studies have correlated recurrent chromosomal translocations distinctive of AML with DNA repair defects. As mentioned earlier, NBS1 mutations expose the genome to a series of risks. A case study has reported that treatment of T-cell NHL in a pediatric NBS patient with DNA topoisomerase II inhibitors has led to a secondary MLL-positive acute monocytic leukemia. This finding suggests that dysfunction of NBS1 may contribute to NHEJ-mediated MLL alterations, especially in patients treated with DNA-damaging agents [68]. In addition, younger age and topoisomerase II inhibitors seem to be implicated in predisposition to t-AML with MLL rearrangements [69]. Oncogenic K-RAS mutations also direct DSB repair in AML cells towards the error-prone A-EJ pathway, and blockage of this pathway could be a potential target in K-RAS mutated cells [56, 70].

Although germline mutations in DSB repair genes are infrequent, transcriptional deregulation and common polymorphisms can predict the patient’s risk to DNA damage and, therefore, the susceptibility to AML development [66]. Compared to mobilized peripheral blood CD34 + progenitor cells from healthy donors, myeloid leukemia cells display elevated activities of error-prone NHEJ and A-EJ pathways [71]. The overexpression of both PARP1 and Lig III markedly favors two or more simultaneous translocations in AML, whereas the patients with one isolated translocation showed overexpression of Lig III alone [72]. AML patients bearing MLL translocations have an intermediate-to-poor prognosis (5-year disease-free survival of 30%-60%), and their leukemia cells are often resistant to conventional chemotherapies. It was shown that PARP1 contributes to the maintenance of MLL-AF9 leukemias. Interestingly, PARP1 inhibition enhances chemosensitivity toward DSB-inducing agents such as cytarabine and doxorubicin in MLL-AF9–positive AML cells [73]. As stated earlier, in FLT3/ITD-positive AML cells, the c-Myc expression is elevated, which in turn contributes to the augmented expression of A-EJ factors, especially PARP1 and Lig III [74]. Strikingly, in FLT3/ITD + cell lines and murine FLT3/ITD bone marrow mononuclear cells, the downregulation of Ku70/80 was coupled with the upregulation of DNA Lig IIIα. Given that FLT3/ITD expression resulted in augmented A-EJ repair, these DNA repair modules constitute appealing targets for developing novel therapeutic approaches in combination with FLT3 inhibitors [75]. SIRT1, a protein directly deacetylating and activating Ku proteins, is another mediator, responsible for the upregulation of C-NHEJ components [76]. Ten-Eleven Translocation-2 (TET2), a member of the TET family of enzymes, has key roles in epigenetic regulation and the occurrence of hematopoietic diseases. It was shown that TET2 overexpression might account for the increased mRNA expression of Lig IV in the HL60 cell line [77, 78]. Likewise, both Lig IV and DNA-PKcs are elevated in daunorubicin (DNR)-resistant HL60 cells [79]. Overall, the upregulation of DSB repair genes facilitates the escape of AML cells from the DNA damage response (DDR) anticancer barrier and causes chemotherapy resistance.

Various polymorphisms in DSB repair genes have been associated with an increased risk of AML development or disease relapse. XRCC1 Arg399Gln and XRCC1 Arg194Trp are the two polymorphic variants of XRCC1 reported in AML patients, associated with downgraded DNA damage repair function [80, 81]. A higher frequency of both XRCC1 polymorphic variants was reported in AML patients. Additionally, both of the variants were also contributed to better overall survival, suggesting that defects in DNA repair elements could influence the predisposition of leukemic cells to chemotherapy treatment [80]. However, Seedhouse et al. observed no correlation between the XRCC1 Arg194Trp genotype and AML/t-AML pathogenesis, and instead, they recognized that XRCC1 Arg399Gln was protecting for t-AML [82]. A meta-analysis study reported no association between XRCC1 polymorphisms and the chance of AML development [83].

#### CML

The leukemic clone of CML originates from a hematopoietic stem cell (HSC) by gaining the chromosomal translocation t(9;22)(q34;q11) containing the BCR-ABL1 fusion gene. CML is characterized by a primary chronic phase that progresses to an accelerated phase and a lethal blast phase [84]. Throughout this course of progress, the activated BCR-ABL1 tyrosine kinase (TK) stimulates various oncogenic pathways (e.g., PI3K/AKT, JAK/STAT), driving malignant differentiation [85]. Therefore, BCR-ABL1 kinase-mediated genetic instability apparently plays a key role in the blastic transformation of CML [86]. SIRT1, an overexpressed protein in CML patients, which can regulate the expression of Ku70 through NHEJ, has a close correlation with the acquisition of BCR-ABL mutations [87]. It was shown that the mechanism involved in the t(9:22) translocation resulting in BCR-ABL1 is frequently due to the SSA and NHEJ [88]. Also, BCR-ABL induces reactive oxygen species (ROS) formation. Subsequently, these species destabilize the genome through unfaithful HR and NHEJ-induced DSBs in proliferating cells [89]. Unfaithful NHEJ-mediated BCR-ABL repair, characterized by the decreased levels of Lig IV and Artemis, but not DNA-PKcs, is compensated by the upregulation of Lig III and WRN proteins [90]. Moreover, by overexpression of c-Myc in leukemic cells, BCR-ABL1 increases the expression of A-EJ factors, including Lig III and PARP1 [74]. K562, a BCR-ABL-harboring cell, shows an increase in WRN and Lig III at the protein level. This overexpression has also been observed in P210MO7e cells, as well as CML patients [91]. Loss of ATM function (even monoallelic loss) was also accelerating the blast crisis in BCR-ABL-expressing CML cells [92]. Overall, the Philadelphia chromosome arises from DSB misrepair through ineffective NHEJ [91, 93].

### Lymphoma

Lymphomas are fundamentally divided into two main groups: Hodgkin lymphoma (HL) and NHL. B-cell NHL frequently exhibits recurrent reciprocal translocations, which commonly involve a juxtaposition of immunoglobulin heavy chain (IgH) loci by a proto-oncogene (e.g., BCL2 and BCL6) [94]. Likewise, the development of HL is partially followed by adverse alleles in base excision repair (BER) and DSBR genes, such as XRCC1, the main factor of MMEJ [95]. Also, the rapid development of lymphoma in Lig IV^−/−^p53^−/−^, XRCC4^−/−^p53^−/−^, Ku80^−/−^p53^−/−^, and DNA-PKcs^−/−^p53^−/−^ mice supports the notion that lymphomagenesis is increased by NHEJ loss, especially if the p53 activity is impaired [96].

Oncogenes sometimes have a direct impact on DSBR or may be indirectly involved in DSBR by affecting the progression of the cell cycle and the production of ROS. Oncogenic expression of RAS and suppression of ATR synergistically increase genomic instability in AML caused by MLL-ENL [97], as well as c-Myc-driven lymphoma [98]. Myc plays a key role in increasing the A-EJ activity in TK-activated leukemia through transcriptional and post-transcriptional changes in Lig III and PARP1 [99]. It is known that c-Myc exerts two paradoxical effects on cancer. First, it induces DDR to recognize and repair the damage through ATM/CHK2, leading to tumor suppression. Second, it modulates replication stress through the ATR/CHK1 pathway and protects cancer cell viability [100].

In diffuse large B-cell lymphoma (DLBCL) cells, the expression of key MMEJ proteins, including Lig I, Lig III, PARP1, CtIP, and MRE11 elevates, while the level of C-NHEJ factors decreases [101]. SUDHL8, a cell line driven from a DLBCL patient, showed the increased expression of XRCC6 by four to five folds and the reduced expression of MRE11 by two folds, compared to benign reactive lymphocytes. This pattern not only can be seen in DLBCL but is also consistently observed in other mature B cell lymphomas, including follicular lymphoma (FL), mantle cell lymphoma (MCL), and marginal zone lymphoma (MZL) [102]. Epstein–Barr virus (EBV)-driven NK/T lymphoma also has a profile of downregulated Cernunnos (XLF) [103].

Mutations in DDR genes, including Artemis, DNA-PKcs, Ku70, Ku80, CHK2, and PARP1, have also been reported in DLBCL [104]. Through inactivation of ARF and p53, two potent tumor suppressor proteins, mutated ATM contributes to tumorigenesis [105]. Besides quantitative mutations, qualitative or functional mutations are also observed in NHEJ factors, including Artemis, DNA-PKcs, XRCC5/Ku80, and XRCC6/Ku70, especially in DLBCL with translocations [104]. MCL, another NHL, refers to an aggressive hematologic malignancy with a poor prognosis. Statistical analysis revealed that 26% of MCL cases had p53 mutation/deletion, 56% showed ATM alterations, and 10% showed both alterations. The p53 mutation status is correlated with the extent of cell response to PARP and ATM targeting [106, 107]. Although ATM alteration is mostly observed in B-CLL, MCL, and T-PLL, it has also been infrequently identified in DLBCL, FL, and rarely, adult ALL [108]. Also, a particular subtype of MCL, leukemic non-nodal MCL, is associated with the deletion of PARP1 [109]. Finally, activation-induced cytidine deaminase (AID), which is responsible for DSB generation in CSR, plays an important role in the generation of Ig-partnered chromosome translocations in many B cell lymphomas and leukemias. Also, AID can be a source of secondary mutations in some types of human cancers, such as ALL and CML, thereby contributing to tumor progression [110].

The presence of T-nucleotides at t(11;14)/CCND1-IgH junction in MCL suggests the involvement of an aberrant V(D)J recombination and NHEJ or A-EJ repair pathways in MCL. A similar finding has also been reported at t(14;18)/IgH-MALT1 in mucosa-associated lymphoid tissue (MALT) lymphoma and at t(14;18)/IGH-BCL2 in FL [111]. Correspondingly, t(11;18)(q21;q21) translocation of MALT lymphoma may be the consequence of aberrant NHEJ following DSB [112].

#### MDS

Several studies have shown that MDS cases are at significance risk of transforming into AML. Various predicting factors, such as mutations in NRAS, KRAS, PTPN11, FLT3-ITD, NPM1, WT1, and IDH2, as well as monosomy 7, complex karyotype, and loss of 17p have been found to be related to MDS transformation into AML [113, 114]. MDS refers to HSC diseases and is characterized by an elevated NHEJ activity [115]. De Laval et al. showed that upon exposure to ionizing radiation, TPO promotes C-NHEJ in stem and progenitor cell populations through binding to its receptor (MPL), thereby initiating MDS; however, this TPO/DNA-PK-mediated NHEJ repair pathway in HSC may be defective [116, 117]. It was shown that downregulation of some NHEJ factors, such as Lig IV, Ku70, and Ku80, are involved in primary MDS [118]. Besides, the expression level of PARP1, an A-EJ factor, has been newly approved as a prognostic factor of MDS. PARP1 mRNA expression was shown to be the only biomarker of response to hypomethylating agents (HMAs) 5-azacytidine in patients with MDS. Patients with higher PARP1 mRNA levels had a better response to 5-azacytidine and longer median survival after treatment initiation, suggesting that PARP1 can potentially serve as a guide to therapeutic decisions [119]. However, it exhibits an inverse correlation with prognosis in AML [120]. Other factors, such as ATM, XRCC6, and Lig IV, are also overexpressed in MDS patients as a consequence of some functional polymorphisms in their germlines [121, 122].

MDS patients, especially patients with late refractory anemia with excess blasts (RAEB-1), exhibit a high expression of phosphorylated ATM, phosphorylated Chk2, and γH2AX, according to the immunostaining analysis [123, 124]. These patients and other high-risk MDS patients have mutations in CtIP and MRE11, which lead to microsatellite instability [125]. These findings not only disclose the role of genomic instability in MDS, but also propose some biomarkers for MDS, as they remarkably accord with γH2AX. The γH2AX level is generally considered a biomarker of DSB and is especially altered in therapy-related MDS (t-MDS). It is known that t-MDS is caused by DSB inducers, such as etoposide, and NHEJ acts as the main route for the repair of etoposide-induced DSB [126]. Collectively, γH2AX and 53BP1 localization in MDS are considered useful biomarkers of the increased level of NHEJ [123].

#### MM

MM is a B cell neoplasm of the bone marrow characterized by various clinical presentations, including anemia, bone lesions, infection, hypercalcemia, and renal insufficiency [127]. Mutations in ATM, ATR, MRN complex, XRCC3, XRCC4, and BRCA1, as well as DDR ubiquitin ligase, RNF168, are continuously reported in MM [128, 129]. Both NHEJ and HR mechanisms have shown to be aberrantly upregulated in myeloma cells. In this regard, Herrero et al. observed the upregulation of DNA-PKcs, Artemis, and XRCC4 in MM. They also reported an upregulation of the A-EJ protein DNA ligase IIIα in plasma cells isolated from patients with MM [130]. Compared to normal B cells, a compelling body of evidence shows that the expression of XRCC6 is downregulated in MM and other lymphoma cells. However, unlike XRCC6, there is an increase in the expression level of XRCC4 in MM patients, compared to mature B cell lymphomas, such as MCL, FCL, and DLBCL [102].

Moreover, the increased expression of XRCC4 and Lig IV has been observed in a melphalan-resistant cell line [131]. There is also an elevation in the expression of XRCC5 and Artemis genes in MM cells, compared to monoclonal gammopathy of unknown significance (MGUS) plasma cells [132]. The expression of other components, such as ERCC1, has recently attracted the researchers’ attention, considering its association with sensitivity to melphalan and cisplatin. Additionally, overactivation of A-EJ components, especially Lig IIIa, has been frequently observed in MM cells [133]. Despite previous reports, knowledge in this field is still limited, and further studies are required. Table 1 summarizes the NHEJ alterations in hematologic malignancies.

**Table 1:** Alterations of NHEJ (classical or alternative) level in hematologic malignancies

Type of malignancy	Subtype of malignancy	Involved factor ↑ = Increase ↓ = Decrease	Highlights	Ref
Leukemia	FLT3/ITD-positive AML	↓ Ku70/80, ↑ PARP-1 and DNA Lig IIIα	Disease progression and Chemoresistance	[75]
	APL	Homozygous variants of BRCA2 and XRCC5	Risk of secondary APL development	[62]
	MLL-rearranged AML	↑ PARP1	Maintenance of MLL-AF9 in Leukemia	[73]
		Coexistence of NBS1 and MLL mutations	Increase chance of secondary malignancy after treatment with DNA topoisomerase II inhibitors	[68]
	K562/DNR	↑ DNA-PKcs and Lig IV	DNR resistant More aggressive MDR phenotype	[79]
	CML	↓ DNA-PK, Lig IV, and Artemis ↑ Lig III and WRN	Progression to blast crisis	[91, 134]
	K562 cells (BCR-ABL^+^)	↑ WRN and Lig III ↓ Artemis	Increased repair infidelity and survival of leukemic cells	[91, 93]
	ALL	Mutations in LIG IV, ATM, and NBS1	Development of disease	[50, 51, 135]
		↑ mRNA of Ku70, Ku80, DNA-PK, Artemis, Lig IV XRCC4, and Cernunnos ↑ 53BP1/γH2AX foci	Unfaithful DSBR and increased genome instability Causing BCR-ABL and TEL-AML fusions	[57, 70, 136]
		Polymorphisms in XRCC6 and XRCC1	Ethnic-dependent increased risk of ALL	[59, 60, 137]
	KRAS-mutant T-ALL	↑ DNA Lig IIIα, PARP1, and XRCC1	Hyperactivation of more error-prone pathways (A-EJs)	[138]
	T-ALL	↑ PI3K/mTOR pathway (ATM-ATR-DNA-PK)	Poor prognosis and failure of treatment	[139]
	CLL	↑ MMEJ factor and DNA-PK	Poor survival of patients	[6, 42]
		Mutation or deletion of ATM	Chemoresistance	[140]
		↑ SSA	Telomere fusion	[45]
Lymphoma	C-MYC-driven lymphoma	↑ ATM/CHK2 ↑ ATR/CHK1	Paradoxical effects, including tumor suppression and protection of the viability of cancer cells	[100]
	Mature B cell lymphoma (FL, MCL, DLBCL, MALT, and MZL)	↑ Lig I, Lig III, PARP1, CtIP, and MRE11 ↓ C-NHEJ functional mutations in Artemis, DNA-PKcs, XRCC5, and XRCC6	High level of DSB and aberrant DSBR	[101, 102]
	DLBCL	Mutations in ATM	Inactivation of ARF as a tumor suppressor gene and P53	[141]
	Leukemic non-nodal MCL	Deletion of PARP1	Unfavourable outcome	[109]
	EBV-driven NK/T lymphoma	↓ Cernunnos (XLF)	Genomic instability	[103]
	HL	Adverse alleles of DSBR genes, including XRCC1	Genomic chaos Dicentric chromosomes resulting from telomere dysfunction and aberrant NHEJ	[142, 143]
MDS	MDS	↑ Defective C-NHEJ ↑ A-EJ ↓ Lig IV, Ku70, and Ku80	A contingently ineffective increase in C-NHEJ	[118, 144]
MM	MM	↑ DNA-PKcs, Artemis, XRCC4, and Lig IIIa ↓ XRCC6 ↑ Lig IV and XRCC4 Mutations in ATM, ATR, MRN complex, XRCC3, and XRCC4	Ineffective increase of C-NHEJ pathway	[77, 128–130]
		ERCC1	Prediction of response to melphalan and cisplatin	[133]

### Treatment of hematologic malignancies by targeting the components of DSBR:

Malignant cells, which are defective in one pathway, are dependent on other pathways; accordingly, many studies have applied a targeting strategy against these pathways. Several studies revealed that repair knockout mouse models display developmental deficiencies, suggesting that repair proteins have numerous functions. In this regard, it should be noticed that the chemical inhibition of repair protein components presents a totally different scenario compared to gene knockouts. Chemical inhibitions are applied in shorter durations and localized manner [145]. Here, we conducted an elaborated review of different inhibitors against NHEJ, which can be used as a treatment strategy for hematologic malignancies. Also, a thorough status of clinical trials of these inhibitors for blood malignancies has been listed in Table 2.

**Table 2:** Overview on clinical trials of hematological malignancies treated with DNA repair inhibitors

Target	Conditions	Compound	Phases	Participants	Status	NCT Number
PAPR	Myelodysplastic Syndrome and Acute Myeloid Leukemia Related to PARP Inhibitors (MyeloRIB)	PARP Inhibitors	–	178	Completed	NCT04326023
	Acute Lymphoblastic Leukemia Acute Myeloid Leukemia	Veliparib (ABT-888) Temozolomide	Phase 1	66	Active, not recruiting	NCT01139970
	Acute Myeloid Leukemia Recurrent Myelodysplastic Syndrome	Olaparib	Phase 2	94	Recruiting	NCT03953898
	Leukemia Lymphoma	veliparib	Phase 1	23	Completed	NCT00387608
	Chronic Lymphocytic Leukemia T-cell-prolymphocytic Leukemia	Niraparib (MK4827)	Phase 1	113	Completed	NCT00749502
	Acute Myeloid Leukemia Myelodysplastic Syndrome Chronic Lymphocytic Leukemia Mantle Cell Lymphoma	BMN-673 (talazoparib)	Phase 1	33	Completed	NCT01399840
	Leukemia	BMN 673	Phase 1	12	Recruiting	NCT03974217
	Acute Myeloid Leukemia	BMN 673 Decitabine	Phase 1 Phase 2	25	Active, not recruiting	NCT02878785
	B-cell Malignancy, Low-grade	E7449 (dual PARP1/2 and TNKS1/2 inhibitor) alone E7449 plus TMZ E7449 plus carboplatin and paclitaxel	Phase 1 Phase 2	41	Completed	NCT01618136
	Adult Acute Megakaryoblastic Leukemia Adult Acute Myeloid Leukemia Chronic Myelomonocytic Leukemia Essential Thrombocythemia Myelodysplastic Syndrome Philadelphia Chromosome Negative, BCR-ABL1 Positive Chronic Myelogenous Leukemia Polycythemia Vera Recurrent Adult Acute Lymphoblastic Leukemia	Veliparib Topotecan-Hydrochloride Carboplatin	Phase 1	12	Active, not recruiting	NCT00588991
	Acute Myeloid Leukemia Atypical Chronic Myeloid Leukemia, BCR-ABL1 Negative| Chronic Myelomonocytic Leukemia| Essential Thrombocythemia| Myelodysplastic/Myeloproliferative Neoplasm| Myelofibrosis| Polycythemia Vera|	Veliparib Topotecan-Hydrochloride Carboplatin	Phase 2	60	Suspended	NCT03289910
	Leukemia| Lymphoma| Waldenstrom Macroglobulinemia|	Veliparib Rituximab Bendamustine- Hydrochloride	Phase 1 Phase 2	43	Completed	NCT01326702
	Mantle Cell Lymphoma	CEP-9722 Gemcitabine Cisplatin	Phase 1	24	Completed	NCT01345357
DNA-PK	Chronic Lymphocytic Leukemia	CC-115	Phase 1	118	Completed	NCT01353625
	Refractory/Recurrent Acute Myeloid Leukemia	MSC2490484A (M3814) Mitoxantrone Etoposide Cytarabine	Phase 1	48	Recruiting	NCT03983824
	Chronic Lymphocytic Leukemia	MSC2490484A (M3814)	Phase 1	31	Completed	NCT02316197
	Lymphoma, Non-Hodgkin	CC-122 (Avadomide)	Phase 1	15	Active, not recruiting	NCT02509039
	Large B-Cell, Diffuse Lymphoma, Non-Hodgkin	CC-122 Obinutuzumab	Phase 1	75	Active, not recruiting	NCT02417285
	Diffuse B-Cell Lymphoma	CC-122 RCHOP	Phase 1	35	Completed	NCT03283202
	Leukemia, Lymphocytic, Chronic, B-Cell	CC-122 Ibrutinib Obinutuzumab	Phase 1 Phase 2	47	Completed	NCT02406742
	Multiple Myeloma Lymphoma, Large B-Cell, Diffuse	CC-122	Phase 1	271	Active, not recruiting	NCT01421524
	Lymphoma, Large B-Cell, Diffuse	CC-122 CC-223 Rituximab CC-292	Phase 1	174	Active, not recruiting	NCT02031419
	Lymphoma, Non-Hodgkin Lymphoma, Large B-Cell, Diffuse Lymphoma, Follicular	CC-122 JCAR017 Durvalumab Ibrutinib CC-220 Relatlimab Nivolumab CC-99282	Phase 1 Phase 2	77	Recruiting	NCT03310619
	Chronic Lymphoproliferative Diseases	GRN163L (Imetelstat)	Phase 1	48	Completed	NCT00124189
	Multiple Myeloma	GRN163L	Phase 1	40	Completed	NCT00718601
	Multiple Myeloma	GRN163L	Phase 1	20	Completed	NCT00594126
	Primary Myelofibrosis Secondary Myelofibrosis Myeloid Malignancies	GRN163L	Phase 2	81	Completed	NCT01731951
	Myelofibrosis (JAK-Inhibitor Treatment resistance)	GRN163L Best Available Therapy (BAT)	Phase 3	320	Recruiting	NCT04576156
	Myelodysplastic Syndromes	GRN163L Placebo	Phase 2 Phase 3	225	Recruiting	NCT02598661
	Multiple Myeloma	GRN163L lenalidomide	Phase 2	13	Completed	NCT01242930
	Essential Thrombocythemia| Polycythemia Vera	GRN163L	Phase 2	20	Completed	NCT01243073
ATR	Lymphomas	BAY1895344	Phase 1	241	Recruiting	NCT03188965
	11q-deleted Relapsed/Refractory Chronic Lymphocytic Leukaemia (CLL)| Prolymphocytic Leukaemia (PLL)| B Cell Lymphomas	AZD6738 (Ceralasertib)	Phase 1	2	Completed	NCT01955668
	Leukemia| Myelodysplastic Syndrome CMML	AZD6738	Phase 1	52	Recruiting	NCT03770429
	Chronic Lymphocytic Leukemia	AZD6738 Acalabrutinib	Phase 1 Phase 2	12	Active, not recruiting	NCT03328273
	Cancers	AZD6738 Gemcitabine	Phase 1	55	Recruiting	NCT03669601
	Relapsed/refractory aggressive Non-Hodgkin's Lymphoma	AZD6738 AZD9150 Acalabrutinib Hu5F9-G4 Rituximab AZD5153	Phase 1	30	Completed	NCT03527147

### PARP1 inhibition

A-EJs are considered the main cause of translocation. PARP1 by initiating A-EJ seems to be associated with chromosomal translocations. PARP1 inhibition can hinder both ionizing radiation (IR)-generated and topoisomerase II inhibitor-generated translocations [146]. Since Pol θ depletion can increase sensitivity to PARP inhibition, it can serve as a biomarker, indicating the extent of cell response to PARP1 inhibitors [147, 148]. Both quiescent and proliferating leukemia cells are sensitive to PARP1 inhibitors. Therefore, leukemia stem cells and progenitor cells involved in leukemia can be therapeutic targets [149].

The combination of FLT3 and PARP1 inhibitors eliminates both quiescent and proliferating FLT3-ITD-positive AML cells [150]. Response to a PARP inhibitor, olaparib (AZD2281, MK-7339), has been evaluated in MCL cells deficient in both ATM and p53 and the cells lacking ATM function alone. The results showed that ATM- and p53-deficient cells are more sensitive than ATM-deficient cells to olaparib, indicating that p53 regulates the response of ATM‐deficient MCL cells to Olaparib [106]. In contrast, PARP1 inhibition by AG14361 in MCL cell lines shows potent cytotoxicity in combination with topotecan in a p53-independent manner [151].

**Fig. 4:**
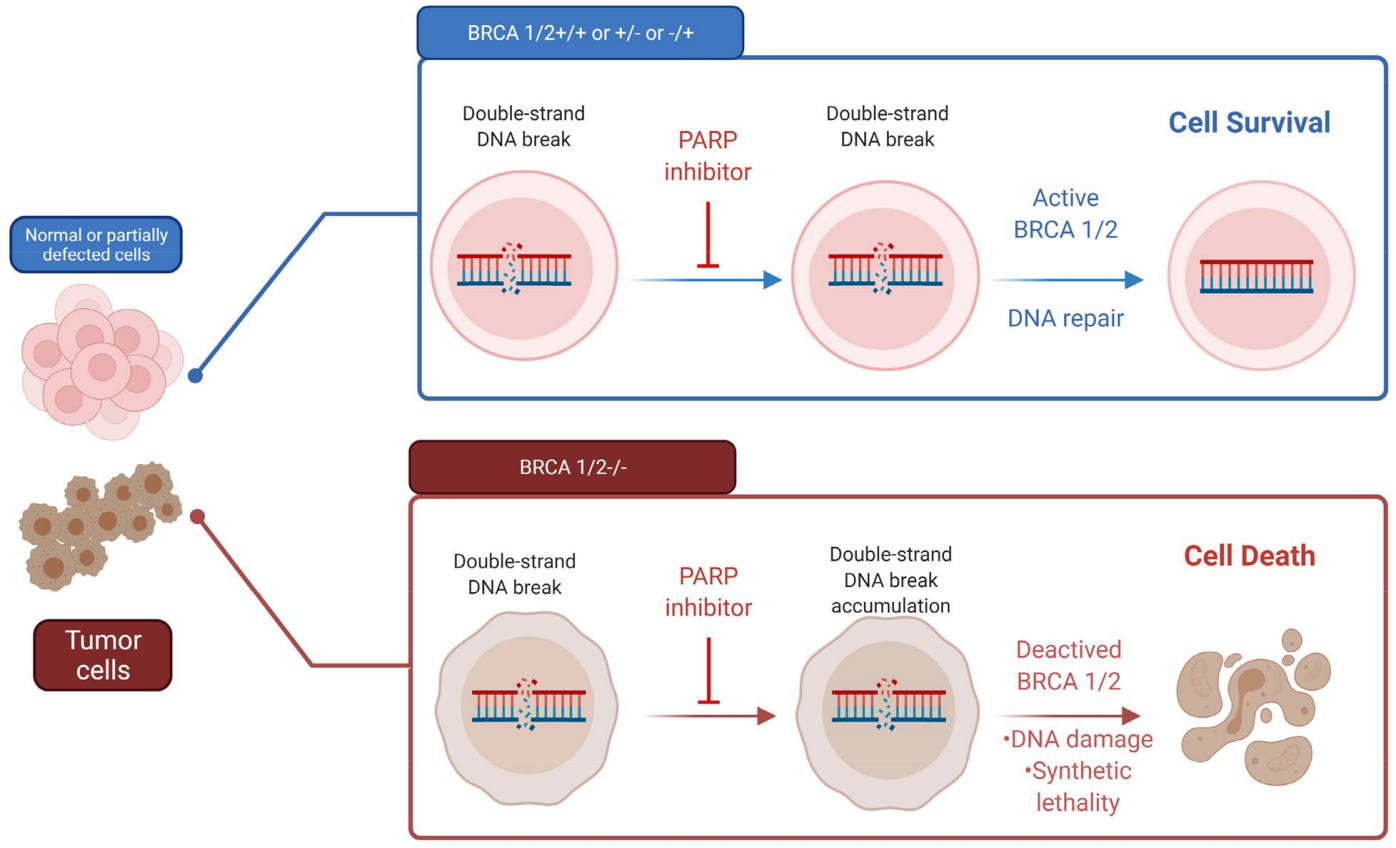
Cells with normal BRCA1/2 or with one normal allele can compensate for double-strand break repair in the presence of PARP inhibitors. On the other hand, using PARP inhibitors in tumor cells with double-negative BRCA1/2 accumulate DSB, which leads to cell death

Tobin et al. demonstrated that the combination of PARP1 with Lig III inhibitors could reduce the survival of CML cells, with the effect being greater in imatinib-resistant CML cells, which express higher levels of PARP1 and Lig III [152]. Moreover, given the remarkable effects of PARP1 inhibitors on the treatment of tumors with decreased levels of BRCA [153], it can be suggested that these inhibitors are beneficial in hematologic malignancies with a reduced BRCA profile, such as CML (Fig. 4) [154].

While some adult T-cell leukemia (ATL) cells are sensitive to PARP inhibitor PJ-34 due to caspase 3-dependent apoptosis, the MT-2 cells (an ATL cell line) are resistant. Augmented expression of BRCA1 or p53-binding protein 1 (P53BP1) has been reported to associate with resistance to PARP inhibitors. However, expression levels of p53BP1 or BRCA1 were not influenced in HTLV-I-transformed MT-2 before or after PJ-34 treatment [155]. PJ-34 has also been shown to be effective in suppressing the proliferation of HL60, MOLT4, and K562 cell lines, but not U937 cells when used in combination with a histone deacetylase inhibitor, vorinostat [156]. The PARP inhibitor also induces synthetic lethality in AML [157]. In a subgroup of AML patients, including those with AML1-ETO translocation, PARP1 inhibitors may be applicable. As mentioned earlier, maintenance of MLL-rearranged AML cells can be a result of PARP1 function. Therefore, PARP1 inhibition by olaparib and talazoparib (BMN-673) in MLL-AF9 leukemia cells increases the number of DSBs, the rate of cell death, and treatment efficacy in combination with conventional therapies [73]. The results of an ex vivo study showed that talazoparib induced a significant inhibitory effect on the proliferation of CLL cells, regardless of the ATM level [158].

Evidence suggests that ATM-deficient tumors are more sensitive to PARP inhibitors. Likewise, ATM-defective CLL cells have a hypersensitive pattern for PARP inhibitors compared to the ATM-proficient counterparts [159]. However, according to some conflicting results, since acetylation inhibits DNA repair factors, and hypomethylation is in favor of hyperacetylation, a combination of a hypomethylation agent with PARP inhibitors can induce apoptosis in human leukemia and lymphoma cells through acetylation of Ku70, Ku80, PARP1, ERCC1, and XPF [160]. Also, veliparib (ABT-888) is a PARP inhibitor with favorable effects against advanced lymphoma and MM when used in combination with bendamustine and rituximab [161].

The results of an in vitro study demonstrated that the combination of ABT-888 with a CDK inhibitor, dinaciclib, is effective in the induction of cell death in MM cells; however, this combined treatment did not exert any cytotoxic effects against normal CD19 + B cells [162]. Phase I trial of the PARP inhibitor veliparib and metronomic cyclophosphamide in patients with low-grade lymphoma showed promising results [163]. Several trials are testing the effectiveness of veliparib in combination with chemotherapeutic drugs, including Phase I trial of ABT-888 with cyclophosphamide and doxorubicin in NHL [ClinicalTrials.gov Identifier: NCT00740805] and phase I trial of ABT-888 with bortezomib and dexamethasone in patients with relapsed refractory myeloma in [ClinicalTrials.gov Identifier: NCT01495351]. On the other hand, a promising response rate to veliparib in combination with topotecan and carboplatin was achieved in patients with aggressive myeloproliferative disorders [164].

Niraparib (MK4827), another PARP1 and PARP2 inhibitor, is in the clinical trial phase for use in monotherapy against CLL and T-PLL [ClinicalTrials.gov Identifier: NCT00749502]. Since MCL is an aggressive malignancy, efforts have been made to find a suitable drug against this disease. CEP-9722 (paralog cep-8983) is also a PARP inhibitor, which is currently in phase I clinical trial for the treatment of MCL in combination with gemcitabine and cisplatin [ClinicalTrials.gov Identifier: NCT01345357]. Talazoparib (BMN-673) inhibits both PARP1 and PARP2 [165]. The effectiveness of talazoparib for the treatment of patients with AML and MDS that have a mutation in the cohesin complex is under investigation in phase I clinical trial [ClinicalTrials.gov Identifier: NCT03974217]. Also, preclinical studies on AML mouse models and primary patient samples revealed that the combination of talazoparib with DNA methyltransferase (DNMT) inhibitor decitabine resulted in enhanced cytotoxicity in AML cells [166].

As mentioned earlier, RNA interference is an interlinking issue in DDR. The overexpression of MALAT1, a long non-coding RNA, plays an important role in DNA repair and cell death in MM cells, especially through interaction with PARP1. MALAT1 degradation by RNase H in MM cells results in poly-ADP-ribosylation of nuclear proteins and further stimulation of apoptotic pathways. Considering the anti-cancer effects of anti-MALAT1 therapy in MM cell lines, xenograft murine models and in vivo models have suggested this agent as a novel therapeutic option against MM [167]. Finally, the novel PARP1 inhibitor, P10, has shown significant effects on the human leukemic cell line, Nalm6, where PARP1 and PARP2 are highly overexpressed [168]. Table 3 summarizes PARP1 targeting in hematologic malignancies.

**Table 3:** Pre-clinical studies on PARP1 and DNA-PK inhibitors against hematologic malignancies

Target	Inhibitors	Type of malignancy	Highlights	Ref
PARP1	Olaparib (AZD2281, MK-7339)	MCL and MLL-AF9 rearranged Leukemia cells	Higher sensitivity of double-deficient ATM/p53 MCL cells, compared to mono-deficient MCL cells in ATM	[106]
	AG14361	MCL cells	Enhanced topotecan-induced apoptosis independent of TP53 status	[151]
	PJ-34	Patient-derived ATLL cells	p53-mediated caspase 3-dependent apoptosis	[155]
		HL60, MOLT4, and K562 human leukemia cell lines	Synergistic effect in combination with histone deacetylase inhibitor, vorinostat	[156]
	BMN-673 (talazoparib)	Patient-derived CLL samples	Inhibited the proliferation of CLL cells independently of p53/ATM function	[158]
		Primary AML samples AML mouse models	Enhanced apoptosis in combination with decitabine	[166]
	Veliparib (ABT-888)	Patients with relapsed/refractory lymphoma and MM	Enhances the cytotoxicity of bendamustine and rituximab	[161]
		MM cells MM xenografts in SCID mice	Combined treatment with CDK inhibitor dinaciclib resulted in synthetic lethality of MM cells	[162]
		Acute leukemia, high-risk MPNs	Promising results in Combined treatment with topotecan and carboplatin in phase I study	[164]
	P10	Human leukemic cell line Nalm6	Induction of G2/M arrest and accumulation of DNA damage	[168]
DNA-PK	Wortmannin	Human leukemia cells	Sonolisib (PX-866) is Irreversible wortmannin analogue PWT-458 is PEGylated derivative of wortmannin	[193, 194]
	NU7026	Primary CLL cells	Synergistic effects with chlorambucil	[195]
	CC-115	CLL, NHL, and MM	Phase I clinical trial A dual inhibitor of DNA-PK and mTOR	[177, 196]
	IC86621	NHL and HL	Synergistic effects with bleomycin and etoposide	[183]
	CC-122 (Avadomide)	NHL and MM	Phase I clinical trial	[178, 197]
	NU7441	Pre-B ALL cells	Increase of chemosensitivity to doxorubicin	[198]
	OK-1035	L5178Y cells (lymphoma cell line)	Preclinical testing	[199]
	Dbait (AsiDNA or DT01)	Lymphoma and leukemia cells	32 bp double-stranded DNA fragment that mimics DNA lesion and traps DNA repair enzymes A dual inhibitor of DNA-PK and PARP1	[191]
	GRN163L (Imetelstat)	CLL	Imetelstat sensitizes primary CLL lymphocytes to fludarabine A dual inhibitor of DNA-PK and telomerase	[192]

### Ku inhibition

Due to the central position of Ku70/80 dimer in NHEJ repair pathways, targeting them seems rational for disrupting the whole pathway. Considering the hyperactivation of the NHEJ pathway in HTLV-1 transformed cells, it looks that targeting Ku70 in these cells can be a suitable therapeutic approach [169]. Since SIRT1 promotes DSBR by deacetylating Ku70 in CML cells, the NHEJ pathway may be impaired through inhibition of SIRT1, which increases Ku70 acetylation [76]. Currently, no small molecule inhibitors against Ku proteins have been developed. However, depletion of Ku70 protein by RNAi technology effectively sensitized the mammary cells to radiation [170–172].

Given the necessity of chromatin remodeling in Ku recruitment, it seems that targeting this process inhibits NHEJ and leads to radio sensitization [173]. The use of HDAC inhibitors, as chromatin remodeling inhibitors, has been approved for patients with refractory cutaneous T-cell lymphoma [174, 175]. In Jurkat T cell lymphoma cells, silencing of Ku70 results in DNA damage accumulation, DDR impairment, reduction of cell proliferation, and induction of cell death; therefore, Ku70 can be a promising target in ATL cells [169].

### DNA-PK inhibition

Inhibition of DNA-PK seems an appealing approach to subside resistance to therapeutically induced DNA DSBs, and for this reason, relatively extensive research has been done in this area. Inhibition of DNA-dependent protein kinases enhances ultrasound-induced apoptosis in human leukemia cell lines U937 and Molt-4, regardless of p53 phenotype, suggesting DNA-PK as a promising target for ultrasound-aided therapy [176]. Critical signaling pathways in CLL are hampered by dual mTOR/DNA-PK inhibition, reducing cell survival and proliferation of chemoresistant CLL cells. CC-115, a dual inhibitor of DNA-PK and mTOR, inhibits proliferation and induces caspase-dependent apoptosis in primary CLL cells. Also, the clinical efficacy of CC-115 was demonstrated in relapsed/refractory CLL/small lymphocytic lymphoma patients harboring ATM deletions/mutations [177]. Also, the effect of CC-122, a DNA-PK inhibitor, in NHL and MM is under investigation, and favorable results have been reported in phase I clinical trial [ClinicalTrials.gov Identifier: NCT01421524] [178].

Deriano et al. demonstrated that NHEJ DSB repair is overactivated in human B-CLL cells in the presence of irradiation-induced DNA damage. This allows the escape of B-CLL cells from apoptosis. Moreover, they showed that NU7026, a DNA-PK inhibitor, can sensitize resistant B-CLL cells to irradiation-induced apoptosis [42]. The growth of MOLT-4 leukemia cells has been reported to be hampered by combination therapy, using NU7026 and radiation [179]. In addition, NU7026 promotes the cytotoxicity of topoisomerase II inhibitors in K562 leukemia cells [180]. The promoting effect of DNA-PK inhibitors on radiation and topoisomerase II inhibitors has been demonstrated in several hematologic cancers, such as CLL, ALL, CML, AML, APL, and adult T-cell leukemia/lymphoma [181]. Given the relationship between ATM deficiency and sensitivity to DNA-PKcs inhibitors, the effects of these inhibitors on lymphoma have been investigated [182].

Bleomycin and etoposide are DSB-inducing agents used against several cancers, especially HL and NHL. Also, IC86621, a selective DNA PK inhibitor, exerts significant synergistic effects when used along with bleomycin and etoposide [183]. Moreover, vanillin as a naturally occurring food component has been shown to have anti-tumor effects, as it can sensitize lymphoblastic TK6 cells to cisplatin through inhibiting the activity of DNA-PK, a crucial NHEJ component [184]. M3814 (MSC2490484A) is another selective DNA-PK inhibitor, which can effectively induce cell death in AML cells by increasing p53-dependent apoptosis [185]. Moreover, the combination of M3814 with Mylotarg (the first AML-targeting drug from a new generation of antibody drug conjugate therapies) in two AML xenograft models, MV4-11 and HL-60, revealed increased efficacy and survival [186]. It should be noted that M3814 is in phase I of a clinical trial for the treatment of CLL patients [ClinicalTrials.gov Identifier: NCT02316197].

Wortmannin is a PI3-kinase inhibitor that also inhibits DNA-PK and thereby impedes DSBs repair [187]. It has been shown that DNA-PK inhibition by wortmannin sensitizes multidrug-resistant (MDR) human leukemia CEM cells (human T-ALL cell line) to chemotherapeutic agents [188]. Akt, a well-known component of the PI3-kinase/Akt/mTOR signaling network, is also a therapeutic target in acute myelogenous leukemia patients and seems to play a role in the phosphorylation of DNA-PK and improving the efficiency of repair [189]. Accordingly, it has been suggested that AKT inhibitors can suppress the phosphorylation of DNA-PK and its activity. Thus, SF-1126, a peptidic pro-drug inhibitor of pan-PI3K/mTORC, has shown satisfactory results against CLL, MM, and NHL in phase I trials [190]. Moreover, PI3K/mTOR overactivation is the cause of relapse in a subtype of pediatric T-ALL; therefore, PKI-587, a dual specificity PI3K/mTOR inhibitor, can be used to inhibit T-ALL cell growth and delay tumor formation [139]. Another novel strategy is to use Dbait (DNA strand break bait) molecules, which mimic DSBs and trap DNA-PK and PARP. Thereby, by generating a false DNA damage signal, they inhibit the recruitment of key repair proteins at the damage site and ultimately prevent the repair of DNA damage. AsiDNA, a cholesterol form of Dbait, exerts synergistic effects in combination with etoposide, cyclophosphamide, and radiotherapy against lymphoma and leukemia cell lines without increasing their toxicity to normal blood cells [191]. GRN163L (Imetelstat; GRN), a 13-mer oligonucleotide complementary to the template of the TER component of telomerase, is a potent telomerase inhibitor. However, it also inhibits DNA-PK activity and repair of DNA damage. In a recent study by Shawi et al., imetelstat was shown to decrease the fludarabine-induced DNA-PK phosphorylation in primary CLL cells [192]. Table 3 presents the potential targeting strategies against DNA-PK in hematologic malignancies.

### ATM inhibition

ATR/ATM kinases are primarily the orchestrators of cellular response to DSB and belong to apical phosphatidylinositol 3-kinase-related kinases (PIKKs). ATM and ATR are predominantly activated through their interactions with NBS1- and RPA-bound single-stranded DNA (ssDNA), respectively [200]. It has been shown that inhibition of ATM and ATR activities promotes survival in xenograft models of AML-carrying MLL rearrangement [201]. KU-55933 was the first developed ATM inhibitor. Mechanistically, KU-55933 impairs the auto-phosphorylation of ATM and concurrently inhibits H2AX phosphorylation. ATM inhibition by KU-55933 sensitized MV4-11 and Jurkat leukemic cells to DSB-inducing agents [202, 203]. It has been shown that inhibition of ATM with two distinct pharmacological inhibitors (namely ATMI and KU55933) induces apoptosis in CD34 + positive leukemic blasts through suppression of constitutively activated NF-κB signaling pathway [204].

Lytic reactivation of EBV in latently infected cells induces an ATM-dependent DDR. Therefore, inhibition of ATM activity by KU-55933 during lytic activation of the virus impairs EBV replication in EBV-infected Burkitt lymphoma cells [205]. The cisplatin-resistant MCL cell line (JeKo-1/DDP) is also affected by KU-55933, causing an increase in cisplatin-induced DNA damage [206]. Finally, ATM inhibition by KU-55933 decreases cell viability in hairy cell leukemia (HCL) cells via inhibiting the hyperactivated NF-κB pathway in these cells [207].

A novel class of ATM inhibitors, known as AZD0156, inhibits ATM kinase activity and exerts similar effects to KU-55933 [208]. This inhibitor produces satisfactory outcomes and shows robust efficacy in murine models of AML [209]. KU-60019 (KU-55933 analog) is a potent and selective inhibitor of ATM, which has been used in the treatment of solid tumors, as well as leukemia and lymphoma [210, 211]. In this regard, KU-60019 potentiates bendamustine activity on human B cell lymphoma cell lines (BALM3, SU-DHL-4, U698M, and SKW4), lymphoblastoid cell line (BALM1), and myeloma cells (RPMI8226) [212].

Caffein can inhibit both ATM and ATR and it induces G1/S checkpoint arrest, as well as a G2/M checkpoint delay in K562 erythroblastic leukemia cells [213]. Nevertheless, similar to wortmannin, the broad nonspecific effects and high in vivo toxicity at the concentrations required to inhibit ATM, prohibit their use in the clinic [214, 215].

### ATR inhibitors

Similar to ATM, ATR inhibition in murine models of MLL-rearranged AML can prevent tumor growth and also reduce the tumor burden. These outcomes have been detected in xenografts of a human AML-MLL cell line [201]. VE-821 is a selective ATR inhibitor, with more than 100-fold selectivity for ATR versus ATM [216]. In combination therapy using ATM inhibitor (KU55933), VE-821 showed an increased radiosensitizing effect in promyelocytic leukemia cell line (HL60) [217]. Similarly, a combination therapy approach, using VE-821 and KU-55933, significantly decreases the survival of MM cells while inhibition of other NHEJ components (i.e., DNA-PK), does not exert any cytotoxic effects on the viability of MM cells [218]. VE-822 (VX-970) is an improved analog of VE-821, which is more soluble, potent, and selective than VE-821 and has better pharmacodynamic properties [219]. In a murine AML model, VE-822 acts as a chemosensitizer in combination with gemcitabine and results in complete eradication of disseminated leukemia [220].

Another ATR inhibitor, AZD6738 (Ceralasertib), is under clinical development and has been approved for oral prescription. It was shown that AZD6738 was selectively cytotoxic to both TP53- and ATM-deficient CLL cell lines and primary tumor samples. Reduction in the proportion of CLL cells was also confirmed in vivo using primary xenograft models of TP53- or ATM-defective CLL. Additionally, AZD6738 sensitized primary CLL cells with such defects to chemotherapy and ibrutinib, suggesting ATR as a promising therapeutic target for TP53- or ATM-defective CLL [221]. A profound synthetic lethal interaction was reported between ATR and the ATM-p53 tumor suppressor pathway in cells treated with DNA-damaging agents [222]. Likewise, inhibition of ATR kinase activity in MCL with ATM-loss of function results in synthetic lethality, which represents ATR inhibitor as a therapeutic approach in ATM-deficient tumors [223]. As a combination therapy, AZD6738 augments carboplatin, bendamustine, and cyclophosphamide effects and reduces the tumor burden in an ATM-deficient DLBCL mouse model [219]. A phase I clinical trial of AZD6738 in combination with acalabrutinib is under evaluation in relapsed or refractory high-risk CLL patients [ClinicalTrials.gov Identifier NCT03328273]. BAY 1895344 is also a novel selective ATR kinase inhibitor. In a panel of cancer cell lines harboring different mutations in DDR pathways, BAY 1895344 displayed potent antiproliferative activity, and MCL cell lines appeared to be the most sensitive cancer type. BAY 1895344 also exhibits a synergistic activity in combination with chemotherapy agents and external beam radiotherapy [224]. At this time, BAY 1895344 is under clinical investigation in patients with advanced solid tumors and lymphomas [ClinicalTrials.gov Identifier: NCT03188965].

Oncogenic expression of Ras and suppression of ATR synergistically increase the genomic instability in MLL-ENL-driven AML, highlighting ATR inhibition as a promising therapeutic strategy. This toxic interaction between ATR suppression and oncogenic stress occurred.

irrespective of status p53 [232]. Treatment with AZ20, another ATR-selective inhibitor, triggered proliferation inhibition in AML cell lines as well as primary patient samples. Moreover, AZ20 synergistically cooperates with cytarabine to generate DNA damage, induce apoptosis, and inhibit proliferation in AML cell lines and primary AML patient samples [233]. Palacin et al. reported that inhibition of the kinase ATR with AZ20 could induce chromosomal breakage and death in a mouse model of MLL-rearranged AML, independently of p53 [201]. Table 4 summarizes ATR and ATM inhibitors in hematologic malignancies.

**Table 4:** Therapeutic approaches against ATR and ATM in hematologic malignancies

Targets	Drugs	Type of malignancy	Highlights	Ref
ATM	KU-55933	Jurkat cells	Impairment of the auto-phosphorylation of ATM and inhibition of H2AX phosphorylation	[203, 225]
		MV4-11 cells (AML cell line)	ATM inhibition radiosensitized MV4-11 leukemia cells	[182]
		P39 and MOLM-13 cell lines (MDS cell lines)	ATM inhibition increase radiosensitization of MDS cells	[182]
		EBV-driven Burkitt lymphoma cells	Inhibition of EBV replication through inhibition of KAP1 phosphorylation	[205]
		Ramos cells	Prevention of ATM auto-phosphorylation and potentiation of etoposide-induced apoptosis	[226]
		Cisplatin-resistant MCL cell line (JeKo-1/DDP)	Enhanced cisplatin-induced DNA damage	[206]
		HCL cell line MLMA	Induction of apoptosis through inhibiting NF-κB pathway	[207]
	KU-59403	Jurkat cells	Showing higher potency, tissue distribution, and efficacy over KU-55933	[225]
	AZD0156	AML	exhibits therapeutic potential in a mouse model of MLL-rearranged AML	[227]
	KU-60019	Human B cell lymphoma cell lines, lymphoblastoid cell lines, and myeloma lines	KU-60019 potentiates bendamustine activity	[212]
		MCL cell lines	KU60019 synergizes the antineoplastic effect of romidepsin	[228]
	Caffeine (Inhibitor of both ATM and ATR)	K562 leukemia cells	Caffeine sensitises the cells to genotoxic modalities, particularly irradiation	[229]
		Lymphoma patients	Potentiated chemotherapy and induction of complete remission	[213]
ATR	VE-821	APL cells	Increase of radio sensitization	[217, 218]
		MM cells	Significantly increased apoptosis of MM cells in combination with KU-55933	[218]
		TP53-mutant MM cell lines	As monotherapy alone and in combination with DNA damaging agents, CX5461 or melphalan	[230]
	VE-822 (VX-970)	AML	Increase antileukemic activity of hydroxyurea and gemcitabine in AML mouse model	[220]
	AZD6738	CLL patients	ATR inhibition induces synthetic lethality in TP53- or ATM-defective CLL cells	[221]
		ATM-deficient DLBCL model	Combination therapy with carboplatin, bendamustine, and cyclophosphamide	[219]
		Relapsed/refractory high-risk CLL patients	A phase I clinical trial of AZD6738 in combination with acalabrutinib [Trial identifier: NCT03328273]	-
	BAY1895344	MCL models	Synergistic anti-tumor activity in combination with DNA damage-inducing chemotherapy or radiation therapy	[224]
	AZ20	AML-MLL murine model	Strong cytotoxic effects in vitro and in murine models, irrespective of p53 status	[231]
	WO2010/073034	ATM-deficient MCL	Promising results both in vitro and in vivo models	[223]

### Lig IV inhibition

SCR7 (an L189 derivative) was initially identified as a DNA ligase IV inhibitor. Srivastava et al. used SCR7 in various cell lines, including human leukemia cells, and found that it could significantly inhibit tumor progression [234]. However, more recent work suggests that this inhibitor is neither a selective nor a potent inhibitor of human DNA ligase IV [235]. The RNA interference strategy against Lig IV leads to significant radio sensitization in multiple cultured cell lines and murine models [172, 236].

### MRN complex inhibition

The MRN complex plays two general and determinative roles in DSB repair: (1) DSB sensitivity by activation of ATM; and (2) determination of the pathway fate by MRE11 nuclease activity. Extended researches on MRE11 resulted in a class of inhibitors that selectively prevent the nuclease activities of MRE11 [237]. This study demonstrated that inhibition of endonuclease activity pushes the cell to NHEJ, and blockading the exonuclease activity causes a repair defect. These observations revealed the therapeutically potential impact of targeting the MRE11. Mirin is a molecule, which inhibits both MRN-dependent activation of ATM and MRE11 exonuclease activity [238]. In c-Myc-driven lymphoma, an increase in DNA damage, reduction of cellular survival, and a sharp increase in the apoptosis rate were seen following the inhibition of MRE11 exonuclease activity. Also, in a murine model with IgH/Myc translocation and c-Myc or N-Myc overexpression, pro-B lymphomagenesis was suppressed by mirin-induced inactivation of MRE11 exonuclease activity [239].

## Conclusion and future prospects

Components of DSBR pathways are the guards of genome integrity. Defects of these components are causal factors for genomic instability, including translocations and DNA mutations which contribute to the development and progression of hematological malignancies. When cancer cells are deficient in certain DNA repair pathway, they are highly addicted to alternative repair pathways for their survival. As a result, identifying the components of these compensating pathways in different types of hematologic malignancies may provide us with potential biomarkers for predicting prognosis and guiding treatment choice. Unfaithful repair of DNA lesions coupled with the survival advantage of tumor cells may contribute to drug resistance in hematologic malignancies. Thus, the application of specific DNA repair targeted agents with DNA damage insult, such as chemotherapy or radiation is a more effective strategy for killing tumor cells.

DNA repair targeted agents are increasingly moving from lab to clinic, which positively affects the treatment opportunities in hematologic malignancies. However, the long-term effects of treatment with DNA repair inhibitors should be evaluated with caution as DNA repair inhibition can compromise genomic integrity in normal cells and potentially may develop a malignant phenotype. Furthermore, resistance to DNA repair inhibitors may be an evolving challenge. Therefore, it is crucial to develop alternative DNA repair targets. On the other hand, the important role of some trace elements [240], cellular processes such as neddylation [241], chromatin remodeling factors [242], and tumor microenvironment [243] on the success of hampering DNA repair pathways is indispensable. For instance, lymphoblasts in ALL overexpress VLA-4, which binds to osteopontin (OPN) secreted by osteoblasts in the bone marrow niche. This interaction of VLA-4 with OPN provides an opportunity for leukemic cells to enter the dormancy phase, which decreases their sensitivity to DSB inducers [244].

Development of clinically validated biomarkers of response and resistance and standard biomarker assays are necessary for the optimization of the clinical application of targeted DNA repair inhibitors. With the great advances made in cancer genomics, we gain better insight into the tumor heterogeneity from patient to patient. Personalized cancer therapy based on a repertoire of DNA repair deficiencies in patients with hematology malignancies can achieve tumor selective therapy and low-side effects. Molecular profiling of tumors will also help clinicians to adjust the dose of chemotherapy in combined-modality strategies in order to reduce the toxicity of current treatments for hematologic malignancies [245, 246].

In summary, this review indicates the potential opportunities to combine C-NHEJ and A-EJ inhibitors with chemoradiation treatment modalities for inducing synthetic lethal vulnerability in hematologic malignant cells with up-regulation of these pathways.

## Data Availability

Not applicable.

## Abbreviations

DSB: DNA double-strand break
CSR: Class-switch recombination
HR: Homologous recombination
NHEJ: Non-homologous end-joining
C-NHEJ: Classical non-homologous end-joining
A-NHEJ or A-EJ: Alternative non-homologous end-joining pathways
AT: Ataxia-telangiectasia
DNA-PK: DNA-dependent protein kinase
DNA-PKcs: DNA-dependent protein kinase catalytic subunit
SSA: Single-strand annealing
MMEJ: Microhomology-mediated end-joining
MDS: Myelodysplastic syndromes
MM: Multiple myeloma
B-CLL: B cell chronic lymphocytic leukemia
PK: DNA–protein kinase
TCRs: T-cell receptors
T-ALL: T-cell ALL
T-PLL: T-cell prolymphocytic leukemia
NHL: Hodgkin lymphoma
ATL: Adult T-cell leukemia
HBZ: Leucine zipper (bZIP) factor
FL: Follicular lymphoma
MCL: Mantle cell lymphoma
MZL: Marginal zone lymphoma
MALT: Mucosa-associated lymphoid tissue
FCL: Follicle center lymphoma
EBV: Epstein–Barr virus
AID: Activation-induced cytidine deaminase
HSC: Hematopoietic stem cell
RAEB-1: Refractory anemia with excess blasts
t-MDS: Therapy-related MDS
MGUS: Monoclonal gammopathy of unknown significance

## References

[1] Cannan WJ, Pederson DS (2016). Mechanisms and consequences of double-strand DNA break formation in chromatin. J Cell Physiol.

[2] Rahimian E, Amini A, Alikarami F, Pezeshki SMS, Saki N, Safa M (2020). DNA repair pathways as guardians of the genome: Therapeutic potential and possible prognostic role in hematologic neoplasms. DNA Repair.

[3] Mehta A, Haber JE (2014). Sources of DNA double-strand breaks and models of recombinational DNA repair. Cold Spring Harb Perspect Biol.

[4] Huang R, Zhou P-K (2021). DNA damage repair: historical perspectives, mechanistic pathways and clinical translation for targeted cancer therapy. Signal Transduct Target Ther.

[5] Frit P, Barboule N, Yuan Y, Gomez D, Calsou P (2014). Alternative end-joining pathway (s): bricolage at DNA breaks. DNA Repair.

[6] Gassner FJ, Schubert M, Rebhandl S, Spandl K, Zaborsky N, Catakovic K, Blaimer S, Hebenstreit D, Greil R, Geisberger R (2018). Imprecision and DNA break repair biased towards incompatible end joining in leukemia. Mol Cancer Res.

[7] Aplan PD (2006). Causes of oncogenic chromosomal translocation. Trends Genet.

[8] Gollin SM (2007). Mechanisms leading to nonrandom, nonhomologous chromosomal translocations in leukemia. Semin Cancer Biol.

[9] Gupta G, Kumar R, Chao H, Simpson D, Kumar S, Wozny A, Purvis J (2020). Hyperactive end joining repair mediates radiation resistance in TP53 deficient cells. Int J Radiat Oncol Biol Phys.

[10] Iliakis G, Wang H, Perrault AR, Boecker W, Rosidi B, Windhofer F, Wu W, Guan J, Terzoudi G, Pantelias G (2004). Mechanisms of DNA double strand break repair and chromosome aberration formation. Cytogenet Genome Res.

[11] Sishc BJ, Davis AJ (2017). The role of the core non-homologous end joining factors in carcinogenesis and cancer. Cancers (Basel).

[12] Scully R, Panday A, Elango R, Willis NA (2019). DNA double-strand break repair-pathway choice in somatic mammalian cells. Nat Rev Mol Cell Biol.

[13] Chang HH, Pannunzio NR, Adachi N, Lieber MR (2017). Non-homologous DNA end joining and alternative pathways to double-strand break repair. Nat Rev Mol Cell Biol.

[14] Davis AJ, Chen DJ (2013). DNA double strand break repair via non-homologous end-joining. Translational cancer research.

[15] Zhao B, Rothenberg E, Ramsden DA, Lieber MR (2020). The molecular basis and disease relevance of non-homologous DNA end joining. Nat Rev Mol Cell Biol.

[16] Waters CA, Strande NT, Wyatt DW, Pryor JM, Ramsden DA (2014). Nonhomologous end joining: a good solution for bad ends. DNA Repair.

[17] Gavande NS, VanderVere-Carozza PS, Hinshaw HD, Jalal SI, Sears CR, Pawelczak KS, Turchi JJJP (2016). therapeutics: DNA repair targeted therapy: the past or future of cancer treatment?. Pharmacol Ther.

[18] Andres SN, Vergnes A, Ristic D, Wyman C, Modesti M, Junop M (2012). A human XRCC4–XLF complex bridges DNA. Nucleic Acids Res.

[19] Boboila C, Yan C, Wesemann DR, Jankovic M, Wang JH, Manis J, Nussenzweig A, Nussenzweig M, Alt FW (2010). Alternative end-joining catalyzes class switch recombination in the absence of both Ku70 and DNA ligase 4. J Exp Med.

[20] Xie A, Kwok A, Scully R (2009). Role of mammalian Mre11 in classical and alternative nonhomologous end joining. Nat Struct Mol Biol.

[21] Mateos-Gomez PA, Gong F, Nair N, Miller KM, Lazzerini-Denchi E, Sfeir A (2015). Mammalian polymerase θ promotes alternative NHEJ and suppresses recombination. Nature.

[22] Boboila C, Jankovic M, Yan CT, Wang JH, Wesemann DR, Zhang T, Fazeli A, Feldman L, Nussenzweig A, Nussenzweig M (2010). Alternative end-joining catalyzes robust IgH locus deletions and translocations in the combined absence of ligase 4 and Ku70. Proc Natl Acad Sci U S A.

[23] Simsek D, Jasin M (2010). Alternative end-joining is suppressed by the canonical NHEJ component Xrcc4–ligase IV during chromosomal translocation formation. Nat Struct Mol Biol.

[24] Bhargava R, Onyango DO, Stark JM (2016). Regulation of single-strand annealing and its role in genome maintenance. Trends Genet.

[25] Sallmyr A, Tomkinson AE (2018). Repair of DNA double-strand breaks by mammalian alternative end-joining pathways. J Biol Chem.

[26] Sallmyr A, Tomkinson AEJ (2018). Repair of DNA double-strand breaks by mammalian alternative end-joining pathways. J Biol Chem.

[27] Hassa PO, Haenni SS, Elser M, Hottiger MO (2006). Nuclear ADP-ribosylation reactions in mammalian cells: where are we today and where are we going?. Microbiol Mol Biol Rev.

[28] Haince J-F, McDonald D, Rodrigue A, Déry U, Masson J-Y, Hendzel MJ, Poirier GG (2008). PARP1-dependent kinetics of recruitment of MRE11 and NBS1 proteins to multiple DNA damage sites. J Biol Chem.

[29] Huertas P (2010). DNA resection in eukaryotes: deciding how to fix the break. Nat Struct Mol Biol.

[30] Kim H-S, Williamson EA, Nickoloff JA, Hromas RA (2017). Metnase mediates loading of exonuclease 1 onto single Strand overhang DNA for end resection at stalled replication forks. J Biol Chem.

[31] Shaheen M, Williamson E, Nickoloff J, Lee SH, Hromas R (2010). Metnase/SETMAR: a domesticated primate transposase that enhances DNA repair, replication, and decatenation. Genetica.

[32] Rath A (2014). Significance of Metnase and Artemis nucleases in determining fidelity ofmammalian end joining repair.

[33] Della-Maria J, Zhou Y, Tsai M-S, Kuhnlein J, Carney JP, Paull TT, Tomkinson AE (2011). Human Mre11/human Rad50/Nbs1 and DNA ligase IIIα/XRCC1 protein complexes act together in an alternative nonhomologous end joining pathway. J Biol Chem.

[34] Thapar R (2018). Regulation of DNA double-strand break repair by non-coding RNAs. Molecules.

[35] Jimeno S, Fernández-Ávila MJ, Cruz-García A, Cepeda-García C, Gómez-Cabello D, Huertas P (2015). Neddylation inhibits CtIP-mediated resection and regulates DNA double strand break repair pathway choice. Nucleic Acids Res.

[36] Ramaekers CH, Wouters BG (2011). Regulatory functions of ubiquitin in diverse DNA damage responses. Curr Mol Med.

[37] Alhmoud JF, Mustafa AG, Malki MI (2020). Targeting DNA repair pathways in hematological malignancies. Int J Mol Sci.

[38] Ahmadi SE, Rahimi S, Zarandi B, Chegeni R, Safa M (2021). MYC: a multipurpose oncogene with prognostic and therapeutic implications in blood malignancies. J Hematol Oncol.

[39] Klein A, Miera O, Bauer O, Golfier S, Schriever F (2000). Chemosensitivity of B cell chronic lymphocytic leukemia and correlated expression of proteins regulating apoptosis, cell cycle and DNA repair. Leukemia.

[40] Holgersson Å, Nilsson A, Lewensohn R, Kanter LJE (2004). Expression of DNA-PKcs and Ku86, but not Ku70, differs between lymphoid malignancies. Exp Mol Pathol.

[41] Willmore E, Elliott SL, Mainou-Fowler T, Summerfield GP, Jackson GH, O'Neill F, Lowe C, Carter A, Harris R, Pettitt AR, Cano-Soumillac C (2008). DNA-dependent protein kinase is a therapeutic target and an indicator of poor prognosis in B-cell chronic lymphocytic leukemia. Clin Cancer Res.

[42] Deriano L, Guipaud O, Merle-Béral H, Binet J-L, Ricoul M, Potocki-Veronese G, Favaudon V, Maciorowski Z, Muller C, Salles B (2005). Human chronic lymphocytic leukemia B cells can escape DNA damage-induced apoptosis through the nonhomologous end-joining DNA repair pathway. Blood.

[43] Popp HD, Flach J, Brendel S, Ruppenthal S, Kleiner H, Seifarth W, Schneider S, Schulze TJ, Weiss C, Wenz F (2019). Accumulation of DNA damage and alteration of the DNA damage response in monoclonal B-cell lymphocytosis and chronic lymphocytic leukemia. Leuk Lymphoma.

[44] Austen B, Powell JE, Alvi A, Edwards I, Hooper L, Starczynski J, Taylor AMR, Fegan C, Moss P, Stankovic T (2005). Mutations in the ATM gene lead to impaired overall and treatment-free survival that is independent of IGVH mutation status in patients with B-CLL. Blood.

[45] Escudero L, Cleal K, Ashelford K, Fegan C, Pepper C, Liddiard K, Baird DM (2019). Telomere fusions associate with coding sequence and copy number alterations in CLL. Leukemia.

[46] Yi M, Zhou L, Li A, Luo S, Wu K (2020). Global burden and trend of acute lymphoblastic leukemia from 1990 to 2017. Aging (Albany NY).

[47] Ding LW, Sun QY, Tan KT, Chien W, Mayakonda A, Yeoh AEJ, Kawamata N, Nagata Y, Xiao JF, Loh XY (2017). Mutational landscape of pediatric acute lymphoblastic leukemia. Cancer Res.

[48] Jafari L, Izadirad M, Vatanmakanian M, Ghaedi H, Farsiani MA, Mohammadi MH, Amiri V, Hosseini MS, Tavakoli F, Gharehbaghian A (2021). IFNG-AS1 and MAF4 long non-coding RNAs are upregulated in acute leukemia patients who underwent bone marrow transplantation. Curr Res Transl Med.

[49] Somsedikova A, Markova E, Kolenova A, Puskacova J, Kubes M, Belyaev I (2014). Constitutive 53BP1/γH2AX foci are increased in cells of ALL patients dependent on BCR-ABL and TEL-AML1 preleukemic gene fusions. Neoplasma.

[50] Ratnaparkhe M, Hlevnjak M, Kolb T, Jauch A, Maass K, Devens F, Rode A, Hovestadt V, Korshunov A, Pastorczak A (2017). Genomic profiling of Acute lymphoblastic leukemia in ataxia telangiectasia patients reveals tight link between ATM mutations and chromothripsis. Leukemia.

[51] Varon R, Reis A, Henze G, Einsiedel HGv, Sperling K, Seeger K (2001). Mutations in the nijmegen breakage syndrome gene (NBS1) in childhood acute lymphoblastic leukemia (ALL). Cancer Res.

[52] Pasic S, Vujic D, Fiorini M, Notarangelo LD (2004). T-cell lymphoblastic leukemia/lymphoma in Nijmegen breakage syndrome. Haematologica.

[53] Dembowska-Baginska B, Perek D, Brozyna A, Wakulinska A, Olczak-Kowalczyk D, Gladkowska-Dura M, Grajkowska W, Chrzanowska KH (2009). Non-Hodgkin lymphoma (NHL) in children with Nijmegen Breakage syndrome (NBS). Pediatr Blood Cancer.

[54] Michallet A-S, Lesca G, Radford-Weiss I, Delarue R, Varet B, Buzyn A (2003). T-cell prolymphocytic leukemia with autoimmune manifestations in Nijmegen breakage syndrome. Ann Hematol.

[55] Riballo E, Critchlow S, Teo S, Doherty A, Priestley A, Broughton B, Kysela B, Beamish H, Plowman N, Arlett C (1999). Identification of a defect in DNA ligase IV in a radiosensitive leukaemia patient. Curr Biol.

[56] Hähnel PS, Enders B, Sasca D, Roos WP, Kaina B, Bullinger L, Theobald M, Kindler T (2014). Targeting components of the alternative NHEJ pathway sensitizes KRAS mutant leukemic cells to chemotherapy. Blood.

[57] Chiou S, Huang J, Tsai Y, Chen T, Lee K, Juo SH, Jong Y, Hung C, Chang T, Lin C (2007). Elevated mRNA transcripts of non-homologous end-joining genes in pediatric acute lymphoblastic leukemia. Leukemia.

[58] Chen TY, Chen JS, Su WC, Wu MS, Tsao CJ (2005). Expression of DNA repair gene Ku80 in lymphoid neoplasm. Eur J Haematol.

[59] Pei J-S, Lee Y-M, Lo H-H, Hsu Y-N, Lin S-S, Bau D-T (2013). Association of X-ray repair cross-complementing-6 genotypes with childhood leukemia. Anticancer Res.

[60] Batar B, Güven M, Barış S, Celkan T, Yıldız İ (2009). DNA repair gene XPD and XRCC1 polymorphisms and the risk of childhood acute lymphoblastic leukemia. Leuk Res.

[61] Meza-Espinoza J, Peralta-Leal V, Gutierrez-Angulo M, Macias-Gomez N, Ayala-Madrigal M, Barros-Nuñez P, Duran-Gonzalez J, Leal-Ugarte E (2009). XRCC1 polymorphisms and haplotypes in Mexican patients with acute lymphoblastic leukemia. Genet Mol Res.

[62] Hasan S, Buttari F, Ottone T, Voso MT, Hohaus S, Marasco E, Mantovani V, Garagnani P, Sanz M, Cicconi L (2011). Risk of acute promyelocytic leukemia in multiple sclerosis: coding variants of DNA repair genes. Neurology.

[63] Wang G, Wang S, Shen Q, Yin S, Li C, Li A, Li J, Zhou J, Liu Q (2009). Polymorphisms in XRCC5, XRCC6, XRCC7 genes are involved in DNA double-strand breaks (DSBs) repair associated with the risk of acute myeloid leukemia (AML) in Chinese population. J Nanjing Med Univ.

[64] Yi M, Li A, Zhou L, Chu Q, Song Y, Wu K (2020). The global burden and attributable risk factor analysis of acute myeloid leukemia in 195 countries and territories from 1990 to 2017: estimates based on the global burden of disease study 2017. J Hematol Oncol.

[65] Yu J, Li Y, Zhang D, Wan D, Jiang Z (2020). Clinical implications of recurrent gene mutations in acute myeloid leukemia. Exp Hematol Oncol.

[66] Esposito MT, So CW (2014). DNA damage accumulation and repair defects in acute myeloid leukemia: implications for pathogenesis, disease progression, and chemotherapy resistance. Chromosoma.

[67] Izadirad M, Jafari L, James AR, Unfried JP, Wu Z-X, Chen Z-S (2021). Long noncoding RNAs have pivotal roles in chemoresistance of acute myeloid leukemia. Drug Discovery Today.

[68] Pastorczak A, Szczepanski T, Trelinska J, Finalet Ferreiro J, Wlodarska I, Mycko K, Polucha A, Sedek L, Meyer C, Marschalek R (2014). Secondary acute monocytic leukemia positive for 11q23 rearrangement in Nijmegen breakage syndrome. Pediatr Blood Cancer.

[69] Armstrong SA, Staunton JE, Silverman LB, Pieters R, den Boer ML, Minden MD, Sallan SE, Lander ES, Golub TR, Korsmeyer SJ (2002). MLL translocations specify a distinct gene expression profile that distinguishes a unique leukemia. Nat Genet.

[70] Nilles N, Fahrenkrog B (2017). Taking a bad turn: compromised DNA damage response in leukemia. Cells.

[71] Gaymes TJ, Mufti GJ (2002). Myeloid leukemias have increased activity of the nonhomologous end-joining pathway and concomitant DNA misrepair that is dependent on the Ku70/86 heterodimer. Cancer Res.

[72] Pashaiefar H, Yaghmaie M, Tavakkoly-Bazzaz J, Ghaffari SH, Alimoghaddam K, Pantea I, Ghavamzadeh AJCJ (2018). The association between PARP1 and LIG3 expression levels and chromosomal translocations in acute myeloid leukemia patients. Cell J.

[73] Maifrede S, Martinez E, Nieborowska-Skorska M, Di Marcantonio D, Hulse M, Le BV, Zhao H, Piwocka K, Tempera I, Sykes SM (2017). MLL-AF9 leukemias are sensitive to PARP1 inhibitors combined with cytotoxic drugs. Blood Adv.

[74] Muvarak N, Kelley S, Robert C, Baer MR, Perrotti D, Gambacorti-Passerini C, Civin C, Scheibner K, Rassool FV (2015). c-MYC generates repair errors via increased transcription of alternative-NHEJ factors, LIG3 and PARP1 tyrosine kinase-activated leukemias. Mol Cancer Res.

[75] Fan J, Li L, Small D, Rassool F (2010). Cells expressing FLT3/ITD mutations exhibit elevated repair errors generated through alternative NHEJ pathways: implications for genomic instability and therapy. Blood.

[76] Zhang W, Wu H, Yang M, Ye S, Li L, Zhang H, Hu J, Wang X, Xu J, Liang A (2016). SIRT1 inhibition impairs non-homologous end joining DNA damage repair by increasing Ku70 acetylation in chronic myeloid leukemia cells. Oncotarget.

[77] Feng Y, Li X, Cassady K, Zou Z, Zhang X (2019). TET2 function in hematopoietic malignancies, immune regulation, and DNA repair. Front Oncol.

[78] Wang Y, Xiao M, Chen X, Chen L, Xu Y, Lv L, Wang P, Yang H, Ma S, Lin H (2015). WT1 recruits TET2 to regulate its target gene expression and suppress leukemia cell proliferation. Mol Cell.

[79] Nishida Y, Mizutani N, Inoue M, Omori Y, Tamiya-Koizumi K, Takagi A, Kojima T, Suzuki M, Nozawa Y, Minami Y (2014). Phosphorylated Sp1 is the regulator of DNA-PKcs and DNA ligase IV transcription of daunorubicin-resistant leukemia cell lines. Biochim Biophys Acta Gene Regul Mech.

[80] Bănescu C, Duicu C, Trifa AP, Dobreanu M (2014). XRCC1 Arg194Trp and Arg399Gln polymorphisms are significantly associated with shorter survival in acute myeloid leukemia. Leuk Lymphoma.

[81] Wang Y, Spitz MR, Zhu Y, Dong Q, Shete S, Wu X (2003). From genotype to phenotype: correlating XRCC1 polymorphisms with mutagen sensitivity. DNA Repair.

[82] Seedhouse C, Bainton R, Lewis M, Harding A, Russell N, Das-Gupta E (2002). The genotype distribution of the XRCC1 gene indicates a role for base excision repair in the development of therapy-related acute myeloblastic leukemia. Blood.

[83] Huang Y, Xie D, Tang N, Wang J, Zeng X, Zhao P, He L (2014). XRCC1 Arg399Gln variation and leukemia susceptibility: evidence from 2,647 cases and 5,518 controls. Tumor Biology.

[84] Ghalesardi OK, Khosravi A, Azizi E, Ahmadi SE, Hajifathali A, Bonakchi H, Shahidi M (2021). The prognostic importance of BCR-ABL transcripts in chronic myeloid leukemia: a systematic review and meta-analysis. Leuk Res.

[85] Houshmand M, Simonetti G, Circosta P, Gaidano V, Cignetti A, Martinelli G, Saglio G, Gale RP (2019). Chronic myeloid leukemia stem cells. Leukemia.

[86] Muvarak N, Nagaria P, Rassool F (2012). Genomic instability in chronic myeloid leukemia: targets for therapy?. Curr Hematol Malig Rep.

[87] Roth M, Wang Z, Chen WYJO (2016). SIRT1 and LSD1 competitively regulate KU70 functions in DNA repair and mutation acquisition in cancer cells. Oncotarget.

[88] Mattarucchi E, Guerini V, Rambaldi A, Campiotti L, Venco A, Pasquali F, Lo Curto F, Porta G (2008). Microhomologies and interspersed repeat elements at genomic breakpoints in chronic myeloid leukemia. Genes Chromosom Cancer.

[89] Nowicki MO, Falinski R, Koptyra M, Slupianek A, Stoklosa T, Gloc E, Nieborowska-Skorska M, Blasiak J, Skorski T (2004). BCR/ABL oncogenic kinase promotes unfaithful repair of the reactive oxygen species–dependent DNA double-strand breaks. Blood.

[90] Sallmyr A, Tomkinson AE, Rassool FV (2008). Up-regulation of WRN and DNA ligase IIIalpha in chronic myeloid leukemia: consequences for the repair of DNA double-strand breaks. Blood.

[91] Sallmyr A, Tomkinson AE, Rassool FV (2008). Up-regulation of WRN and DNA ligase IIIα in chronic myeloid leukemia: consequences for the repair of DNA double-strand breaks. Blood.

[92] Takagi M, Sato M, Piao J, Miyamoto S, Isoda T, Kitagawa M, Honda H, Mizutani S (2013). ATM-dependent DNA damage-response pathway as a determinant in chronic myelogenous leukemia. DNA Repair (Amst).

[93] Burke B, Carroll M (2010). BCR–ABL: a multi-faceted promoter of DNA mutation in chronic myelogeneous leukemia. Leukemia.

[94] Blombery PA, Wall M, Seymour JF (2015). The molecular pathogenesis of B-cell non-Hodgkin lymphoma. Eur J Haematol.

[95] Monroy CM, Cortes AC, Lopez M, Rourke E, Etzel CJ, Younes A, Strom SS, El-Zein R (2011). Hodgkin lymphoma risk: role of genetic polymorphisms and gene–gene interactions in DNA repair pathways. Mol Carcinog.

[96] Sharpless NE, Ferguson DO, O'Hagan RC, Castrillon DH, Lee C, Farazi PA, Alson S, Fleming J, Morton CC, Frank K (2001). Impaired nonhomologous end-joining provokes soft tissue sarcomas harboring chromosomal translocations, amplifications, and deletions. Mol Cell.

[97] Schoppy DW, Ragland RL, Gilad O, Shastri N, Peters AA, Murga M, Fernandez-Capetillo O, Diehl JA, Brown EJ (2012). Oncogenic stress sensitizes murine cancers to hypomorphic suppression of ATR. J Clin Investig.

[98] Gilad O, Nabet BY, Ragland RL, Schoppy DW, Smith KD, Durham AC, Brown EJ (2010). Combining ATR suppression with oncogenic Ras synergistically increases genomic instability, causing synthetic lethality or tumorigenesis in a dosage-dependent manner. Can Res.

[99] Nagaria P, Rassool FV, Curtin N, Pollard J (2018). Alternative non-homologous end-joining: mechanisms and targeting strategies in cancer. Targeting the DNA damage response for anti-cancer therapy.

[100] Campaner S, Amati B (2012). Two sides of the Myc-induced DNA damage response: from tumor suppression to tumor maintenance. Cell Div.

[101] Gopalakrishnan V, Dahal S, Radha G, Sharma S, Raghavan SC, Choudhary B (2019). Characterization of DNA double-strand break repair pathways in diffuse large B cell lymphoma. Mol Carcinog.

[102] Roddam PL, Allan JM, Dring AM, Worrillow LJ, Davies FE, Morgan GJ (2010). Non-homologous end-joining gene profiling reveals distinct expression patterns associated with lymphoma and multiple myeloma. Br J Haematol.

[103] Somasundaram N, Lim JQ, Ong CK, Lim ST (2019). Pathogenesis and biomarkers of natural killer T cell lymphoma (NKTL). J Hematol Oncol.

[104] de Miranda NF, Peng R, Georgiou K, Wu C, Sörqvist EF, Berglund M, Chen L, Gao Z, Lagerstedt K, Lisboa S (2013). DNA repair genes are selectively mutated in diffuse large B cell lymphomas. J Exp Med.

[105] Grønbæk K, Worm J, Ralfkiaer E, Ahrenkiel V, Hokland P, Guldberg P (2002). ATM mutations are associated with inactivation of the ARF-TP53 tumor suppressor pathway in diffuse large B-cell lymphoma. Blood.

[106] Williamson CT, Kubota E, Hamill JD, Klimowicz A, Ye R, Muzik H, Dean M, Tu L, Gilley D, Magliocco AM (2012). Enhanced cytotoxicity of PARP inhibition in mantle cell lymphoma harbouring mutations in both ATM and p53. EMBO Mol Med.

[107] Greiner TC, Dasgupta C, Ho VV, Weisenburger DD, Smith LM, Lynch JC, Vose JM, Fu K, Armitage JO, Braziel RM (2006). Mutation and genomic deletion status of ataxia telangiectasia mutated (ATM) and p53 confer specific gene expression profiles in mantle cell lymphoma. Proc Natl Acad Sci U S A.

[108] Takagi M (2017). DNA damage response and hematological malignancy. Int J Hematol.

[109] Gallo M, Cacheux V, Vincent L, Bret C, Tempier A, Guittard C, Macé A, Leventoux N, Costes V, Szablewski V (2016). Leukemic non-nodal mantle cell lymphomas have a distinct phenotype and are associated with deletion of PARP1 and 13q14. Virchows Arch.

[110] Kumar R, DiMenna LJ, Chaudhuri J, Evans T (2014). Biological function of activation-induced cytidine deaminase (AID). Biomed J.

[111] Penas EMM, Callet-Bauchu E, Ye H, Gazzo S, Berger F, Schilling G, Albert-Konetzny N, Vettorazzi E, Salles G, Wlodarska I (2010). The t (14; 18)(q32; q21)/IGH-MALT1 translocation in MALT lymphomas contains templated nucleotide insertions and a major breakpoint region similar to follicular and mantle cell lymphoma. Blood.

[112] Liu H, Hamoudi RA, Ye H, Ruskone-Fourmestraux A, Dogan A, Isaacson PG, Du MQ (2004). t (11; 18)(q21; q21) of mucosa-associated lymphoid tissue lymphoma results from illegitimate non-homologous end joining following double strand breaks. Br J Haematol.

[113] Paridar M, Zibara K, Ahmadi SE, Khosravi A, Soleymani M, Azizi E, Ghalesardi OK (2021). Clinico-Hematological and cytogenetic spectrum of adult myelodysplastic syndrome: the first retrospective cross-sectional study in Iranian patients. Mol Cytogenet.

[114] Yu J, Li Y, Li T, Li Y, Xing H, Sun H, Sun L, Wan D, Liu Y, Xie X (2020). Gene mutational analysis by NGS and its clinical significance in patients with myelodysplastic syndrome and acute myeloid leukemia. Exp Hematol Oncol.

[115] Thwaites M, Koropatnick J, Tremblay G, O'Connor-McCourt M (2017). AVID200: a novel TGF-β inhibitor for the treatment of anemia associated with myelodysplastic syndromes. Blood.

[116] De Laval B, Pawlikowska P, Barbieri D, Besnard-Guerin C, Cico A, Kumar R, Gaudry M, Baud V, Porteu F (2014). Thrombopoietin promotes NHEJ DNA repair in hematopoietic stem cells through specific activation of Erk and NF-κB pathways and their target, IEX-1. Blood.

[117] de Laval B, Pawlikowska P, Petit-Cocault L, Bilhou-Nabera C, Aubin-Houzelstein G, Souyri M, Pouzoulet F, Gaudry M, Porteu F (2013). Thrombopoietin-increased DNA-PK-dependent DNA repair limits hematopoietic stem and progenitor cell mutagenesis in response to DNA damage. Cell Stem Cell.

[118] Joshi D, Korgaonkar S, Shanmukhaiah C, Vundinti BR (2017). Down regulation of DNA repair genes Lig4, Ku70, Ku80, XRCC3 in primary myelodysplastic syndromes. Meta Gene.

[119] Diamantopoulos PT, Kontandreopoulou C-N, Symeonidis A, Kotsianidis I, Pappa V, Galanopoulos A, Vassilakopoulos T, Dimou M, Solomou E, Kyrtsonis M-C (2019). Bone marrow PARP1 mRNA levels predict response to treatment with 5-azacytidine in patients with myelodysplastic syndrome. Ann Hematol.

[120] Li X, Li C, Jin J, Wang J, Huang J, Ma Z, Huang X, He X, Zhou Y, Xu Y (2018). High PARP-1 expression predicts poor survival in acute myeloid leukemia and PARP-1 inhibitor and SAHA-bendamustine hybrid inhibitor combination treatment synergistically enhances anti-tumor effects. EBioMedicine.

[121] Junior HLR, Maia ARS, Costa MB, Farias IR, de Paula BD, de Oliveira RTG, de Sousa JC, Magalhães SMM, Pinheiro RF (2016). Influence of functional polymorphisms in DNA repair genes of myelodysplastic syndrome. Leuk Res.

[122] Ribeiro HL, de Oliveira RTG, Maia ARS, Pires Ferreira Filho LI, de Sousa JC, Heredia FF, Magalhães SMM, Pinheiro RF (2015). Polymorphisms of DNA repair genes are related to the pathogenesis of myelodysplastic syndrome. Hematol Oncol.

[123] Popp HD, Naumann N, Brendel S, Henzler T, Weiss C, Hofmann W-K, Fabarius A (2017). Increase of DNA damage and alteration of the DNA damage response in myelodysplastic syndromes and acute myeloid leukemias. Leuk Res.

[124] Horibe S, Takagi M, Unno J, Nagasawa M, Morio T, Arai A, Miura O, Ohta M, Kitagawa M, Mizutani S (2007). DNA damage check points prevent leukemic transformation in myelodysplastic syndrome. Leukemia.

[125] Gaymes TJ, Mohamedali AM, Patterson M, Matto N, Smith A, Kulasekararaj A, Chelliah R, Curtin N, Farzaneh F, Shall S (2013). Microsatellite instability induced mutations in DNA repair genes CtIP and MRE11 confer hypersensitivity to poly (ADP-ribose) polymerase inhibitors in myeloid malignancies. Haematologica.

[126] Montecucco A, Zanetta F, Biamonti G (2015). Molecular mechanisms of etoposide. EXCLI J.

[127] Bird SA, Boyd K (2019). Multiple myeloma: an overview of management. Palliat Care Soc Pract.

[128] Neri P, Bahlis NJ (2013). Genomic instability in multiple myeloma: mechanisms and therapeutic implications. Expert Opin Biol Ther.

[129] Cagnetta A, Lovera D, Grasso R, Colombo N, Canepa L, Ballerini F, Calvio M, Miglino M, Gobbi M, Lemoli R (2015). Mechanisms and clinical applications of genome instability in multiple myeloma. BioMed Res Int.

[130] Herrero AB, San Miguel J, Gutierrez NC (2015). Deregulation of DNA double-strand break repair in multiple myeloma: implications for genome stability. PLoS ONE.

[131] Sousa MM, Zub KA, Aas PA, Hanssen-Bauer A, Demirovic A, Sarno A, Tian E, Liabakk NB, Slupphaug G (2013). An inverse switch in DNA base excision and strand break repair contributes to melphalan resistance in multiple myeloma cells. PLoS ONE.

[132] Calimeri T, Fulciniti M, Lin J, Samur MK, Calkins AS, Vahia AV, Pal J, Cea M, Cagnetta A, Cottini F (2012). Aberrant non-homologous end joining in multiple myeloma: a role in genomic instability and as potential prognostic marker. Blood.

[133] Gourzones-Dmitriev C, Kassambara A, Sahota S, Rème T, Moreaux J, Bourquard P, Hose D, Pasero P, Constantinou A, Klein B (2013). DNA repair pathways in human multiple myeloma: role in oncogenesis and potential targets for treatment. Cell Cycle.

[134] Burke BA, Carroll M (2010). BCR-ABL: a multi-faceted promoter of DNA mutation in chronic myelogeneous leukemia. Leukemia.

[135] Altmann T, Gennery AR (2016). DNA ligase IV syndrome; a review. Orphanet J Rare Dis.

[136] Rushing AW, Hoang K, Polakowski N, Lemasson I, Simon V (2018). The human T-cell leukemia virus type 1 basic leucine zipper factor attenuates repair of double-stranded dna breaks via nonhomologous end joining. J Virol.

[137] Sorour A, Ayad MW, Kassem H (2013). The genotype distribution of the XRCC1, XRCC3, and XPD DNA repair genes and their role for the development of acute myeloblastic leukemia. Genet Test Mol Biomarkers.

[138] Hähnel PS, Enders B, Sasca D, Roos WP, Kaina B, Bullinger L, Theobald M, Kindler TJB (2014). Targeting components of the alternative NHEJ pathway sensitizes KRAS-mutant leukemic cells to chemotherapy. Blood.

[139] Gazi M, Moharram SA, Marhäll A, Kazi JU (2017). The dual specificity PI3K/mTOR inhibitor PKI-587 displays efficacy against T-cell acute lymphoblastic leukemia (T-ALL). Cancer Lett.

[140] Sampath D, Plunkett W (2007). The role of DNA repair in chronic lymphocytic leukemia pathogenesis and chemotherapy resistance. Curr Oncol Rep.

[141] Ruhe M, Rabe D, Jurischka C, Schröder J, Schierack P, Deckert PM, Rödiger S (2019). Molecular biomarkers of DNA damage in diffuse large-cell lymphoma—a review. J Lab Precis Med.

[142] Ramos S, Navarrete-Meneses P, Molina B, Cervantes-Barragán DE, Lozano V, Gallardo E, Marchetti F, Frias S (2018). Genomic chaos in peripheral blood lymphocytes of Hodgkin's lymphoma patients one year after ABVD chemotherapy/radiotherapy. Environ Mol Mutagen.

[143] Salas C, Niembro A, Lozano V, Gallardo E, Molina B, Sanchez S, Ramos S, Carnevale A, Pérez-Vera P, Rivera Luna R (2012). Persistent genomic instability in peripheral blood lymphocytes from Hodgkin lymphoma survivors. Environ Mol Mutagen.

[144] Zhou T, Chen P, Gu J, Bishop AJ, Scott LM, Hasty P, Rebel VI (2015). Potential relationship between inadequate response to DNA damage and development of myelodysplastic syndrome. Int J Mol Sci.

[145] Srivastava M, Raghavan SC (2015). DNA double-strand break repair inhibitors as cancer therapeutics. Chem Biol.

[146] Byrne M, Wray J, Reinert B, Wu Y, Nickoloff J, Lee SH, Hromas R, Williamson E (2014). Mechanisms of oncogenic chromosomal translocations. Ann N Y Acad Sci.

[147] Motegi A, Masutani M, Yoshioka K-I, Bessho T (2019). Aberrations in DNA repair pathways in cancer and therapeutic significances. Semin Cancer Biol.

[148] Ceccaldi R, Liu JC, Amunugama R, Hajdu I, Primack B, Petalcorin MI, O’Connor KW, Konstantinopoulos PA, Elledge SJ, Boulton SJ (2015). Homologous-recombination-deficient tumours are dependent on Polθ-mediated repair. Nature.

[149] Nieborowska-Skorska M, Sullivan K, Dasgupta Y, Podszywalow-Bartnicka P, Hoser G, Maifrede S, Martinez E, Di Marcantonio D, Bolton-Gillespie E, Cramer-Morales K (2017). Gene expression and mutation-guided synthetic lethality eradicates proliferating and quiescent leukemia cells. J Clin Investig.

[150] Maifrede S, Nieborowska-Skorska M, Sullivan-Reed K, Dasgupta Y, Podszywalow-Bartnicka P, Le BV, Solecka M, Lian Z, Belyaeva EA, Nersesyan A (2018). Tyrosine kinase inhibitor–induced defects in DNA repair sensitize FLT3 (ITD)-positive leukemia cells to PARP1 inhibitors. Blood.

[151] Golla RM, Li M, Shen Y, Ji M, Yan Y, Fu K, Greiner TC, McKeithan TW, Chan WC (2012). Inhibition of poly (ADP-ribose) polymerase (PARP) and ataxia telangiectasia mutated (ATM) on the chemosensitivity of mantle cell lymphoma to agents that induce DNA strand breaks. Hematol Oncol.

[152] Tobin LA, Robert C, Rapoport AP, Gojo I, Baer MR, Tomkinson AE, Rassool FV (2013). Targeting abnormal DNA double-strand break repair in tyrosine kinase inhibitor-resistant chronic myeloid leukemias. Oncogene.

[153] Bast RC, Mills GB (2010). Personalizing therapy for ovarian cancer: BRCAness and beyond. J Clin Oncol.

[154] Podszywalow-Bartnicka P, Wolczyk M, Kusio-Kobialka M, Wolanin K, Skowronek K, Nieborowska-Skorska M, Dasgupta Y, Skorski T, Piwocka K (2014). Downregulation of BRCA1 protein in BCR-ABL1 leukemia cells depends on stress-triggered TIAR-mediated suppression of translation. Cell Cycle.

[155] Bai XT, Moles R, Chaib-Mezrag H, Nicot C (2015). Small PARP inhibitor PJ-34 induces cell cycle arrest and apoptosis of adult T-cell leukemia cells. J Hematol Oncol.

[156] Jasek E, Gajda M, Lis GJ, Jasińska M, Litwin JA (2014). Combinatorial effects of PARP inhibitor PJ34 and histone deacetylase inhibitor vorinostat on leukemia cell lines. Anticancer Res.

[157] Zhao L, So CWE (2016). PARP-inhibitor-induced synthetic lethality for acute myeloid leukemia treatment. Exp Hematol.

[158] Herriott A, Tudhope SJ, Junge G, Rodrigues N, Patterson MJ, Woodhouse L, Lunec J, Hunter JE, Mulligan EA, Cole M (2015). PARP1 expression, activity and ex vivo sensitivity to the PARP inhibitor, talazoparib (BMN 673), in chronic lymphocytic leukaemia. Oncotarget.

[159] Weston VJ, Oldreive CE, Skowronska A, Oscier DG, Pratt G, Dyer MJ, Smith G, Powell JE, Rudzki Z, Kearns P (2010). The PARP inhibitor olaparib induces significant killing of ATM-deficient lymphoid tumor cells in vitro and in vivo. Blood.

[160] Valdez BC, Li Y, Murray D, Liu Y, Nieto Y, Champlin RE, Andersson BS (2018). Combination of a hypomethylating agent and inhibitors of PARP and HDAC traps PARP1 and DNMT1 to chromatin, acetylates DNA repair proteins, down-regulates NuRD and induces apoptosis in human leukemia and lymphoma cells. Oncotarget.

[161] Soumerai JD, Zelenetz AD, Moskowitz CH, Palomba ML, Hamlin PA, Noy A, Straus DJ, Moskowitz AJ, Younes A, Matasar MJ (2017). The PARP inhibitor veliparib can be safely added to bendamustine and rituximab and has preliminary evidence of activity in B-cell lymphoma. Clin Cancer Res.

[162] Alagpulinsa DA, Ayyadevara S, Yaccoby S, Shmookler Reis RJ (2016). A cyclin-dependent kinase inhibitor, dinaciclib, impairs homologous recombination and sensitizes multiple myeloma cells to PARP inhibition. Mol Cancer Ther.

[163] Kummar S, Ji J, Morgan R, Lenz HJ, Puhalla SL, Belani CP, Gandara DR, Allen D, Kiesel B, Beumer JH (2012). A phase I study of veliparib in combination with metronomic cyclophosphamide in adults with refractory solid tumors and lymphomas. Clin Cancer Res.

[164] Pratz KW, Rudek MA, Gojo I, Litzow MR, McDevitt MA, Ji J, Karnitz LM, Herman JG, Kinders RJ, Smith BD (2017). A phase I study of topotecan, carboplatin and the PARP inhibitor veliparib in acute leukemias, aggressive myeloproliferative neoplasms, and chronic myelomonocytic leukemia. Clin Cancer Res.

[165] Shen Y, Rehman FL, Feng Y, Boshuizen J, Bajrami I, Elliott R, Wang B, Lord CJ, Post LE, Ashworth A (2013). BMN 673, a novel and highly potent PARP1/2 inhibitor for the treatment of human cancers with DNA repair deficiency. Clin Cancer Res.

[166] Muvarak NE (2017). Developing a novel combination therapy and elucidating mechanisms of increased ALT-NHEJ in acute myeloid leukemia.

[167] Hu Y, Lin J, Fang H, Fang J, Li C, Chen W, Liu S, Ondrejka S, Gong Z, Reu F (2018). Targeting the MALAT1/PARP1/LIG3 complex induces DNA damage and apoptosis in multiple myeloma. Leukemia.

[168] Hegde M, Mantelingu K, Swarup HA, Pavankumar CS, Qamar I, Raghavan SC, Rangappa KS (2016). Novel PARP inhibitors sensitize human leukemic cells in an endogenous PARP activity dependent manner. RSC Adv.

[169] Yu W, Li L, Wang G, Zhang W, Xu J, Liang A (2018). KU70 inhibition impairs both non-homologous end joining and homologous recombination DNA damage repair through SHP-1 induced dephosphorylation of SIRT1 in adult T-cell leukemia-lymphoma cells. Cell Physiol Biochem.

[170] Gu Y, Jin S, Gao Y, Weaver DT, Alt FW (1997). Ku70-deficient embryonic stem cells have increased ionizing radiosensitivity, defective DNA end-binding activity, and inability to support V (D) J recombination. Proc Natl Acad Sci.

[171] Vandersickel V, Mancini M, Slabbert J, Marras E, Thierens H, Perletti G, Vral A (2010). The radiosensitizing effect of Ku70/80 knockdown in MCF10A cells irradiated with X-rays and p (66)+ Be (40) neutrons. Radiat Oncol.

[172] Ratnayake G, Bain AL, Fletcher N, Howard CB, Khanna KK, Thurecht KJ (2018). RNA interference to enhance radiation therapy: targeting the DNA damage response. Cancer Lett.

[173] Oike T, Ogiwara H, Amornwichet N, Nakano T, Kohno T (2014). Chromatin-regulating proteins as targets for cancer therapy. J Radiat Res.

[174] Duvic M, Talpur R, Ni X, Zhang C, Hazarika P, Kelly C, Chiao JH, Reilly JF, Ricker JL, Richon VM (2007). Phase 2 trial of oral vorinostat (suberoylanilide hydroxamic acid, SAHA) for refractory cutaneous T-cell lymphoma (CTCL). Blood.

[175] Piekarz RL, Frye R, Turner M, Wright JJ, Allen SL, Kirschbaum MH, Zain J, Prince HM, Leonard JP, Geskin LJ (2009). Phase II multi-institutional trial of the histone deacetylase inhibitor romidepsin as monotherapy for patients with cutaneous T-cell lymphoma. J Clin Oncol.

[176] Furusawa Y, Fujiwara Y, Hassan MA, Tabuchi Y, Morita A, Enomoto A, Kondo T (2012). Inhibition of DNA-dependent protein kinase promotes ultrasound-induced cell death including apoptosis in human leukemia cells. Cancer Lett.

[177] Thijssen R, Ter Burg J, Garrick B, van Bochove GG, Brown JR, Fernandes SM, Rodriguez MS, Michot JM, Hallek M, Eichhorst B (2016). Dual TORK/DNA-PK inhibition blocks critical signaling pathways in chronic lymphocytic leukemia. Blood.

[178] Rasco DW, Papadopoulos KP, Pourdehnad M, Gandhi AK, Hagner PR, Li Y, Wei X, Chopra R, Hege K, DiMartino J (2019). A first-in-human study of novel cereblon modulator avadomide (CC-122) in advanced malignancies. Clin Cancer Res.

[179] Tichý A, Novotná E, Durisová K, Salovská B, Sedlaríková R, Pejchal J, Zárybnická L, Vávrová J, Sinkorová Z, Rezácová M (2012). Radio-sensitization of human leukaemic molt-4 cells by DNA-dependent protein kinase inhibitor, NU7026. Acta Medica (Hradec Kralove).

[180] Willmore E, de Caux S, Sunter NJ, Tilby MJ, Jackson GH, Austin CA, Durkacz BW (2004). A novel DNA-dependent protein kinase inhibitor, NU7026, potentiates the cytotoxicity of topoisomerase II poisons used in the treatment of leukemia. Blood.

[181] Hsu FM, Zhang S, Chen BP (2012). Role of DNA-dependent protein kinase catalytic subunit in cancer development and treatment. Transl Cancer Res.

[182] Knittel G, Rehkämper T, Nieper P, Schmitt A, Flümann R, Reinhardt HC (2018). DNA damage pathways and B-cell lymphomagenesis. Curr Opin Hematol.

[183] Kashishian A, Douangpanya H, Clark D, Schlachter ST, Eary CT, Schiro JG, Huang H, Burgess LE, Kesicki EA, Halbrook J (2003). DNA-dependent protein kinase inhibitors as drug candidates for the treatment of cancer. Mol Cancer Ther.

[184] Durant S, Karran P (2003). Vanillins—a novel family of DNA-PK inhibitors. Nucleic Acids Res.

[185] Haines, E., Zimmermann, A., Zenke, F., Blaukat, A., & Vassilev, L. T. Selective DNA-PK inhibitor, M3814, boosts p53 apoptotic response to DNA double strand breaks and effectively kills acute leukemia cells: Implications for AML therapy. Cancer Res. 2018; pp 4830-4830

[186] Carr MI, Zimmermann A, Chiu LY, Zenke FT, Blaukat A, Vassilev LT (2020). DNA-PK inhibitor, M3814, as a new combination partner of mylotarg in the treatment of acute myeloid leukemia. Front Oncol.

[187] Powis G, Bonjouklian R, Berggren MM, Gallegos A, Abraham R, Ashendel C, Zalkow L, Matter WF, Dodge J, Grindey G (1994). Wortmannin, a potent and selective inhibitor of phosphatidylinositol-3-kinase. Cancer Res.

[188] Kim SH, Um JH, Dong-Won B, Kwon BH, Kim DW, Chung BS, Kang CD (2000). Potentiation of chemosensitivity in multidrug-resistant human leukemia CEM cells by inhibition of DNA-dependent protein kinase using wortmannin. Leuk Res.

[189] Martelli AM, Evangelisti C, Chiarini F, McCubrey JA (2010). The phosphatidylinositol 3-kinase/Akt/mTOR signaling network as a therapeutic target in acute myelogenous leukemia patients. Oncotarget.

[190] Mahadevan D, Chiorean EG, Harris WB, Von Hoff DD, Stejskal-Barnett A, Qi W, Anthony SP, Younger AE, Rensvold DM, Cordova F (2012). Phase I pharmacokinetic and pharmacodynamic study of the pan-PI3K/mTORC vascular targeted pro-drug SF1126 in patients with advanced solid tumours and B-cell malignancies. Eur J Cancer.

[191] Thierry S, Jdey W, Alculumbre S, Soumelis V, Noguiez-Hellin P, Dutreix M (2017). The DNA repair inhibitor Dbait is specific for malignant hematologic cells in blood. Mol Cancer Ther.

[192] Shawi M, Chu TW, Martinez-Marignac V, Yu Y, Gryaznov SM, Johnston JB, Lees-Miller SP, Assouline SE, Autexier C, Aloyz R (2013). Telomerase contributes to fludarabine resistance in primary human leukemic lymphocytes. PLoS ONE.

[193] Kuete V, Saeed ME, Kadioglu O, Börtzler J, Khalid H, Greten HJ, Efferth T (2015). Pharmacogenomic and molecular docking studies on the cytotoxicity of the natural steroid wortmannin against multidrug-resistant tumor cells. Phytomedicine.

[194] Kim S-H, Um J-H, Kim D-W, Kwon B-H, Kim D-W, Chung B-S, Kang C-D (2000). Potentiation of chemosensitivity in multidrug-resistant human leukemia CEM cells by inhibition of DNA-dependent protein kinase using wortmannin. Leuk Res.

[195] Amrein L, Loignon M, Goulet AC, Dunn M, Jean-Claude B, Aloyz R, Panasci L (2007). Chlorambucil cytotoxicity in malignant B lymphocytes is synergistically increased by 2-(morpholin-4-yl)-benzo[h]chomen-4-one (NU7026)-mediated inhibition of DNA double-strand break repair via inhibition of DNA-dependent protein kinase. J Pharmacol Exp Ther.

[196] Munster P, Mita M, Mahipal A, Nemunaitis J, Massard C, Mikkelsen T, Cruz C, Paz-Ares L, Hidalgo M, Rathkopf D (2019). First-in-human phase I study of a dual mTOR kinase and DNA-PK inhibitor (CC-115) in advanced malignancy. Cancer Manag Res.

[197] Carpio C, Bouabdallah R, Ysebaert L, Sancho JM, Salles G, Cordoba R, Pinto A, Gharibo M, Rasco D, Panizo C (2020). Avadomide monotherapy in relapsed/refractory DLBCL: safety, efficacy, and a predictive gene classifier. Blood.

[198] Alikarami F, Safa M, Faranoush M, Hayat P, Kazemi A (2017). Inhibition of DNA-PK enhances chemosensitivity of B-cell precursor acute lymphoblastic leukemia cells to doxorubicin. Biomed Pharmacother.

[199] Durisova K, Salovska B, Pejchal J, Tichy A (2016). Chemical inhibition of DNA repair kinases as a promising tool in oncology. Biomed Pap Med Fac Univ Palacky Olomouc.

[200] Larsen DH, Stucki M (2016). Nucleolar responses to DNA double-strand breaks. Nucleic Acids Res.

[201] Morgado-Palacin I, Day A, Murga M, Lafarga V, Anton ME, Tubbs A, Chen H-T, Ergen AV, Anderson R, Bhandoola AJSS (2016). Targeting the kinase activities of ATR and ATM exhibits antitumoral activity in mouse models of MLL-rearranged AML. Sci Signal.

[202] Boehrer S, Ades L, Tajeddine N, Hofmann WK, Kriener S, Bug G, Ottmann OG, Ruthardt M, Galluzzi L, Fouassier C (2009). Suppression of the DNA damage response in acute myeloid leukemia versus myelodysplastic syndrome. Oncogene.

[203] Korwek Z, Sewastianik T, Bielak-Zmijewska A, Mosieniak G, Alster O, Moreno-Villanueva M, Burkle A, Sikora E (2012). Inhibition of ATM blocks the etoposide-induced DNA damage response and apoptosis of resting human T cells. DNA Repair (Amst).

[204] Grosjean-Raillard J, Tailler M, Ades L, Perfettini JL, Fabre C, Braun T, De Botton S, Fenaux P, Kroemer G (2009). ATM mediates constitutive NF-kappaB activation in high-risk myelodysplastic syndrome and acute myeloid leukemia. Oncogene.

[205] Hau PM, Tsao SW (2017). Epstein-barr virus hijacks DNA damage response transducers to orchestrate its life cycle. Viruses.

[206] Yan W, Yang Y, Yang W (2019). Inhibition of SKP2 activity impaired ATM-mediated DNA repair and enhanced sensitivity of cisplatin-resistant mantle cell lymphoma cells. Cancer Biother Radiopharm.

[207] Nagel S, Ehrentraut S, Meyer C, Kaufmann M, Drexler HG, MacLeod RA (2015). NFkB is activated by multiple mechanisms in hairy cell leukemia. Genes Chromosom Cancer.

[208] Riches LC, Trinidad AG, Hughes G, Jones GN, Hughes AM, Thomason AG, Gavine P, Cui A, Ling S, Stott J (2020). Pharmacology of the ATM inhibitor AZD0156: potentiation of irradiation and olaparib responses preclinically. Mol Cancer Ther.

[209] Morgado-Palacin I, Day A, Murga M, Lafarga V, Anton ME, Tubbs A, Chen HT, Ergan A, Anderson R, Bhandoola A (2016). Targeting the kinase activities of ATR and ATM exhibits antitumoral activity in mouse models of MLL-rearranged AML. Sci Signal.

[210] Vecchio D, Daga A, Carra E, Marubbi D, Raso A, Mascelli S, Nozza P, Garre ML, Pitto F, Ravetti JL (2015). Pharmacokinetics, pharmacodynamics and efficacy on pediatric tumors of the glioma radiosensitizer KU60019. Int J Cancer.

[211] Inamdar AA, Goy A, Ayoub NM, Attia C, Oton L, Taruvai V, Costales M, Lin YT, Pecora A, Suh KS (2016). Mantle cell lymphoma in the era of precision medicine-diagnosis, biomarkers and therapeutic agents. Oncotarget.

[212] Inoue M, Honma Y, Urano T, Suzumiya J (2017). Japanese apricot extract (MK615) potentiates bendamustine-induced apoptosis via impairment of the DNA damage response in lymphoma cells. Oncol Lett.

[213] Hayashi M, Tsuchiya H, Yamamoto N, Karita M, Shirai T, Nishida H, Takeuchi A, Tomita K (2005). Caffeine-potentiated chemotherapy for metastatic carcinoma and lymphoma of bone and soft tissue. Anticancer Res.

[214] Karve S, Werner ME, Sukumar R, Cummings ND, Copp JA, Wang EC, Li C, Sethi M, Chen RC, Pacold ME (2012). Revival of the abandoned therapeutic wortmannin by nanoparticle drug delivery. Proc Natl Acad Sci U S A.

[215] Newton R, Broughton L, Lind M, Morrison P, Rogers H, Bradbrook I (1981). Plasma and salivary pharmacokinetics of caffeine in man. Eur J Clin Pharmacol.

[216] Fokas E, Prevo R, Pollard JR, Reaper PM, Charlton PA, Cornelissen B, Vallis KA, Hammond EM, Olcina MM, Gillies McKenna W (2012). Targeting ATR in vivo using the novel inhibitor VE-822 results in selective sensitization of pancreatic tumors to radiation. Cell Death Dis.

[217] Vávrová J, Zárybnická L, Lukášová E, Řezáčová M, Novotná E, Šinkorová Z, Tichý A, Pejchal J, Ďurišová K (2013). Inhibition of ATR kinase with the selective inhibitor VE-821 results in radiosensitization of cells of promyelocytic leukaemia (HL-60). Radiat Environ Biophys.

[218] Herrero AB, Gutiérrez NC (2017). Targeting ongoing DNA damage in multiple myeloma: effects of DNA damage response inhibitors on plasma cell survival. Front Oncol.

[219] Karnitz LM, Zou L (2015). Molecular pathways: targeting ATR in cancer therapy. Clin Cancer Res.

[220] Fordham SE, Blair HJ, Elstob CJ, Plummer R, Drew Y, Curtin NJ, Heidenreich O, Pal D, Jamieson D, Park C (2018). Inhibition of ATR acutely sensitizes acute myeloid leukemia cells to nucleoside analogs that target ribonucleotide reductase. Blood Adv.

[221] Kwok M, Davies N, Agathanggelou A, Smith E, Oldreive C, Petermann E, Stewart G, Brown J, Lau A, Pratt GJB (2016). ATR inhibition induces synthetic lethality and overcomes chemoresistance in TP53-or ATM-defective chronic lymphocytic leukemia cells. Blood.

[222] Reaper PM, Griffiths MR, Long JM, Charrier J-D, MacCormick S, Charlton PA, Golec JM, Pollard JR (2011). Selective killing of ATM-or p53-deficient cancer cells through inhibition of ATR. Nat Chem Biol.

[223] Menezes DL, Holt J, Tang Y, Feng J, Barsanti P, Pan Y, Ghoddusi M, Zhang W, Thomas G, Holash J (2015). A synthetic lethal screen reveals enhanced sensitivity to ATR inhibitor treatment in mantle cell lymphoma with ATM loss-of-function. Mol Cancer Res.

[224] Wengner AM, Siemeister G, Lucking U, Lefranc J, Wortmann L, Lienau P, Bader B, Bomer U, Moosmayer D, Eberspacher U (2020). The novel ATR inhibitor BAY 1895344 is efficacious as monotherapy and combined with DNA damage-inducing or repair-compromising therapies in preclinical cancer models. Mol Cancer Ther.

[225] Batey MA, Zhao Y, Kyle S, Richardson C, Slade A, Martin NM, Lau A, Newell DR, Curtin NJ (2013). Preclinical evaluation of a novel ATM inhibitor, KU59403, in vitro and in vivo in p53 functional and dysfunctional models of human cancer. Mol Cancer Ther.

[226] Phan RT, Saito M, Kitagawa Y, Means AR, Dalla-Favera R (2007). Genotoxic stress regulates expression of the proto-oncogene Bcl6 in germinal center B cells. Nat Immunol.

[227] Prakash A, Garcia-Moreno J, Brown J, Bourke E (2018). Clinically applicable inhibitors impacting genome stability. Molecules.

[228] Scotto L, Jirau-Serrano X, Zullo K, Mangone M, Amengual JE, Deng C, O'Connor OA (2015). The ATM inhibitor KU60019 synergizes the antineoplastic effect of romidepsin in mantle cell lymphoma (MCL). Blood.

[229] Sarkaria JN, Busby EC, Tibbetts RS, Roos P, Taya Y, Karnitz LM, Abraham RT (1999). Inhibition of ATM and ATR kinase activities by the radiosensitizing agent, caffeine. Cancer Res.

[230] Bouard L, Tessoulin B, Descamps G, Touzeau C, Moreau P, Amiot M, Pellat-Deceunynck C (2019). Inhibition of ATR overcomes chemotherapy resistance in p53 deficient myeloma cells. Blood.

[231] Kwok M, Stankovic T: Targeting the Ataxia Telangiectasia and Rad3 Signaling Pathway to Overcome Chemoresistance in Cancer. In: Targeting Cell Survival Pathways to Enhance Response to Chemotherapy*.* Elsevier; 2019: 203–230. 10.1016/B978-0-12-813753-6.00010-X

[232] Schoppy DW, Ragland RL, Gilad O, Shastri N, Peters AA, Murga M, Fernandez-Capetillo O, Diehl JA, Brown EJ (2012). Oncogenic stress sensitizes murine cancers to hypomorphic suppression of ATR. J Clin Invest.

[233] Ma J, Li X, Su Y, Zhao J, Luedtke DA, Epshteyn V, Edwards H, Wang G, Wang Z, Chu R (2017). Mechanisms responsible for the synergistic antileukemic interactions between ATR inhibition and cytarabine in acute myeloid leukemia cells. Sci Rep.

[234] Srivastava M, Nambiar M, Sharma S, Karki SS, Goldsmith G, Hegde M, Kumar S, Pandey M, Singh RK, Ray P (2012). An inhibitor of nonhomologous end-joining abrogates double-strand break repair and impedes cancer progression. Cell.

[235] Greco GE, Matsumoto Y, Brooks RC, Lu Z, Lieber MR, Tomkinson AE (2016). SCR7 is neither a selective nor a potent inhibitor of human DNA ligase IV. DNA Repair (Amst).

[236] Frank KM, Sekiguchi JM, Seidl KJ, Swat W, Rathbun GA, Cheng H-L, Davidson L, Kangaloo L, Alt FW (1998). Late embryonic lethality and impaired V (D) J recombination in mice lacking DNA ligase IV. Nature.

[237] Shibata A, Moiani D, Arvai AS, Perry J, Harding SM, Genois M-M, Maity R, van Rossum-Fikkert S, Kertokalio A, Romoli F (2014). DNA double-strand break repair pathway choice is directed by distinct MRE11 nuclease activities. Mol Cell.

[238] Dupré A, Boyer-Chatenet L, Sattler RM, Modi AP, Lee J-H, Nicolette ML, Kopelovich L, Jasin M, Baer R, Paull TT (2008). A forward chemical genetic screen reveals an inhibitor of the Mre11–Rad50–Nbs1 complex. Nat Chem Biol.

[239] Capper, Kayla. Targeting DNA Repair Mechanisms in MYC-Driven Cancer. The University of Michigan. PhD dissertation. 2017. https://deepblue.lib.umich.edu/handle/2027.42/137016

[240] Samavarchi Tehrani S, Mahmoodzadeh Hosseini H, Yousefi T, Abolghasemi M, Qujeq D, Maniati M, Amani J (2018). The crosstalk between trace elements with DNA damage response, repair, and oxidative stress in cancer. J Cell Biochem.

[241] Paiva C, Godbersen J, Berger A, Brown J, Danilov A (2015). Targeting neddylation induces DNA damage and checkpoint activation and sensitizes chronic lymphocytic leukemia B cells to alkylating agents. Cell Death Dis.

[242] Stadler J, Richly H (2017). Regulation of DNA repair mechanisms: how the chromatin environment regulates the DNA damage response. Int J Mol Sci.

[243] Klein TJ, Glazer PM (2010). The tumor microenvironment and DNA repair. Semin Radiat Oncol.

[244] Evans EB, Lin SY (2015). New insights into tumor dormancy: targeting DNA repair pathways. World J Clin Oncol.

[245] Dorgalaleh A, Bahraini M, Ahmadi SE, Dabbagh A (2021). Personalized anesthesia in hematology. Personalized medicine in anesthesia, pain and perioperative medicine.

[246] Hayden EC (2009). Personalized cancer therapy gets closer. Nature.

